# Multimaterial Digital Light Processing Three-Dimensional Printing of Materials with Different Relaxation Kinetics

**DOI:** 10.1021/acsapm.5c02906

**Published:** 2025-10-30

**Authors:** Roman Korotkov, Milena Gleirscher, Sandra Schlögl, Elisabeth Rossegger

**Affiliations:** 1 226178 Polymer Competence Center Leoben GmbH, Sauraugasse 1, Leoben 8700, Austria; 2 Institute for Chemistry and Technology of Materials, 27253 Graz University of Technology, Stremayrgasse 9/V, Graz 8010, Austria

**Keywords:** multimaterial, DLP 3D printing, covalent adaptable network, stress relaxation, modeling

## Abstract

Covalent adaptable networks (CANs) are cross-linked polymer networks, which are able to undergo topological rearrangements due to dynamic exchange reactions under an external stimulus (typically heat), allowing these materials to be malleable, weldable, and recyclable. The temperature-dependent kinetics of dynamic exchange reactions can be tuned by varying the concentration of the dynamic links. However, decreasing the number of dynamic linkages increases the impact of diffusion factors, causing deviation from the Arrhenius-like dependence. Herein, we study the exchange dynamics in the photocurable thiol–ene CANs relying on the thiol-thioester exchange mechanism and investigate the proportion of Arrhenius behavior and Rouse diffusion on the overall stress relaxation kinetics. Materials differing substantially in their stress relaxation kinetics are printed via dual-vat multimaterial DLP 3D printing, and the creep performance of the multimaterial samples is evaluated. In addition, these multimaterial samples can be used as simple temperature–time sensors based on thermal imprinting.

## Graphical abstract

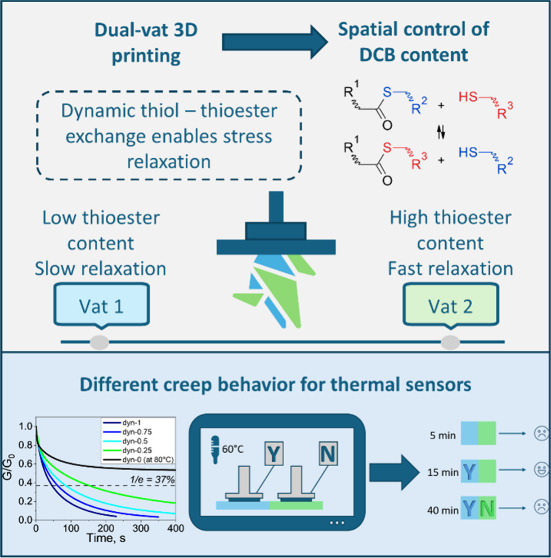

## Introduction

Nowadays, one of the key objectives of research on polymer materials is to meet the challenges of a green and sustainable future. A promising class of sustainable polymers is covalent adaptable networks (CANs), which serve as a bridge between classical thermosetting and thermoplastic polymers.^1^ These materials are characterized by a cross-linked network structure that can undergo a topological rearrangement through bond exchange reactions under certain stimuli, such as heat, light, etc.^2–4^ Depending on the type of bond exchange mechanism, CANs can be divided into two major groups: dissociative and associative.^4–8^ In dissociative networks, bond breakage is followed by bond formation, which leads to a reduction of the cross-link density and a drop in viscosity that can even result in the loss of structural integrity. In associative CANs, bond formation takes place first, maintaining the average cross-link density. At a sufficiently high bond exchange rate, the networks become malleable, weldable, and recyclable.^9–13^ First works on dissociative CANs, utilizing Diels–Alder chemistry, were published in the early 2000s. Associative CANs were introduced in the early 2010s, describing dynamic epoxy-acid and epoxy-anhydride networks, which undergo thermo-activated transesterification reactions.^14,15^ Over the past years, many different types of associative dynamic bond exchange reactions have been investigated.^16–19^

Especially, extensive research is being conducted in the field of 3D printing of CANs as this method allows the production of objects possessing geometries that are difficult to manufacture using conventional techniques. The use of CANs benefits from traditional thermosets, as these materials allow the self-healing and recycling of 3D-printed objects.^20–23^ Among various 3D printing methods, vat photopolymerization (VPP) techniques, including digital light processing (DLP) and stereolithography (SLA), enable the production of thermoset materials with high resolution and excellent mechanical robustness.^24^ DLP 3D printing relies on the selective curing of liquid photopolymer resins by using a digital projector as the light source, which initiates polymerization and cures entire cross sections simultaneously, layer by layer. The introduction of multimaterial 3D printing enables additional spatial control over the properties of 3D-printed objects, such as programming their dynamic behavior,^25,26^ tuning mechanical properties, or incorporating various colors^27^ within a single 3D-printed part.^28^ By employing a dual-wavelength approach, orthogonal photochemistries are utilized to produce materials with varying mechanical properties.^29^ For 3D printing of CANs, our group previously employed this approach to form a hybrid dynamic–permanent network by spatially activating a photolatent transesterification catalyst.^26,30^ Alternatively, using different resins in separate vats also allows the creation of 3D-printed objects with tunable mechanical properties.^31,32^ Although this approach requires an additional cleaning step when changing materials, it enables the combination of multiple material systems within a single printing process. Beyond enhancing circularity, the use of CANs facilitates the incorporation of additional functionalities into 3D-printed objects, as it opens new possibilities for altering the shape, properties, and functionality of printed structures—a concept referred to as 4D printing, denoting 3D printing plus time-dependent change in properties.^33,34^

At present, extensive research is focusing on controlling the rate of exchange reactions of CANs. Adjusting the kinetics of topological rearrangement ensures a target stress relaxation behavior and thus optimum performance in terms of creep resistance, operating temperature, and recyclability.^35^ Depending on the chemistry of the dynamic exchange reactions, the rate can be tuned by varying the stiffness of the network, type and loading of the catalyst, and the concentration of the dynamic groups involved.^15,36,37^ However, an increasing bond exchange rate usually leads to a higher creep, even at lower temperatures, which is not desired for various applications. Therefore, achieving better creep resistance while maintaining recycability and reshapeability still remains challenging.^38–40^

One aspect of the research in this area focuses on varying the concentration of dynamic cross-links. Li et al. showed that a reduction in dynamic bond content can be used to control the creep in thiol-epoxy associative CANs relying on transesterification reactions.^41^ The authors defined a theoretical threshold of dynamic linkages up to which the recyclability of the material remains at an efficient level. Breuillac et al. showed the possibility of implementing this approach to polybutadiene rubbers cross-linked with static and dynamic dioxoborolane cross-linkers^42^ to reduce creep and control the stress relaxation kinetics. The Ramis group investigated the dependence of the topological rearrangement kinetics on the concentration of the dynamic and permanent cross-links in thiol–ene networks based on disulfide metathesis and transesterification exchange mechanism.^43,44^

One of the main factors affected by varying the concentration of dynamic cross-links is the distance between reactive sides.^45^ At a certain distance, the diffusion factor starts to play a significant role in the kinetics of exchange reactions. The diffusion is also influenced by the cross-linking density,^46–48^ concentration of the free functional groups participating in the exchange reactions,^37,49^ concentration of the catalyst^15,50–52^ and the network structure itself.^35,40,53,54^ Isogai et al. demonstrated that the functionality of the cross-linker has an impact on the relaxation behavior in CANs relying on transesterification reactions.^55^ The position of the dynamic cross-link also plays an important role. In CANs based on dioxoborolane metathesis, faster exchange kinetics were observed between cross-links and groups in dangling chains than between the cross-links themselves.^56^

The stress relaxation behavior of CANs can be characterized by analyzing the time-dependent normalized stress decay, which is an essential tool for further modeling and evaluation of the material performance.^57^ In ideal CANs, the stress relaxation properties are expressed with the simple Maxwell model.^30,58^ However, in networks exhibiting nonideal behavior, more complex models are needed.^59^ The stretched exponential Kohlrausch–Williams–Watts (KWW) equation is used to express the deviation from an ideal behavior with the implementation of the stretch factor β in the range from 0 to 1, where smaller β values indicate a larger deviation from the ideal behavior.^60,61^ For networks possessing several bond exchange mechanisms, the multimodal Maxwell model can be applied.^62^

The obtained data is used to determine the characteristic stress relaxation time, an extremely important factor in calculating the activation energy of the process and the topological freezing temperature (*T_v_*).^13^ The stress relaxation time is inversely proportional to the rate to the rate of the topological rearrangement reactions.^38,63^ As long as the main influence on the relaxation rate is the dynamic bond exchange, it follows the Arrhenius law. However, if other factors, such as diffusion, play a role, the rate of these mechanisms becomes crucial in the characterization of the material flow behavior.^61,63^

Herein, we investigate the effect of a variation in the concentration of the dynamic thioester bonds on the exchange dynamics in a photocurable thiol–ene system relying on the thiol-thioester exchange mechanism, which possesses fast dynamic exchange kinetics.^64–66^ The relative contribution of Arrhenius behavior and Rouse diffusion to the overall stress relaxation kinetics was investigated using the Kohlrausch–Williams–Watts (KWW) stretched exponential function with a particular focus on assessing the role of diffusion. The different stress relaxation kinetics of the studied materials allowed for dual-vat multimaterial DLP 3D printing of objects, exhibiting advanced creep behavior. The combination of the static and dynamic resins allowed the 3D printing of cubes possessing the anisotropic creep behavior only in one direction. Moreover, the multimaterial samples were utilized as a simple temperature–time sensor based on thermal imprinting.

## Materials and Methods

### Materials

All chemicals were used as received without additional purification. Pentaerythritol tetrakis­(3-mercaptopropionate) (PETMP, 95%+) was kindly provided by Bruno Bock (Marschacht, Germany). Ethyl (2,4,6-trimethylbenzoyl) phenylphosphinate (TPO-L, 98%+) was obtained from IGM Resins B.V. (Waalwijk, Netherlands). Diallyladipate (DAA, 98%+) was supplied by TCI Europe (Haven, Belgium). Pyridine (anhydrous, 99.8%, <0.003% water), succinic anhydride (for synthesis, 98%+), 3-mercaptopropionic acid (for synthesis, 98%+), 4-(dimethylamino) pyridine (for synthesis, 99%+), para-toluenesulfonic acid monohydrate (TsOH, ACS reagent grade, 98.5%+), 1,1,3,3,-tetramethylguanidine (TMG, 99%+), 1-(2,4-xylylazo)-2-naphthol (Sudan II, dye content 90%) and allyl alcohol (99.0%) were supplied by Sigma-Aldrich (St. Louis, USA). Ethyl acetate (99.5%+), cyclohexane (for synthesis, 99.5%+), anhydrous acetonitrile (MeCN, 99.9%+, ≤10 ppm of H_2_O), isopropanol (99.9%+), toluene (for synthesis, 99.5%+), and anhydrous sodium sulfate (Na_2_SO_4_, 98.5%+) were purchased from Carl Roth (Karlsruhe, Germany).

### Synthesis of Thioester Monomer

The synthesis of the thioester monomer TE-2 was performed according to the literature with small adaptations.^65^

### Synthesis of TE-1

In a 2 L round-bottomed flask equipped with a magnetic stirrer, 900 mL of anhydrous MeCN and 100 mL of anhydrous pyridine (9:1 ratio) were placed. Subsequently, succinic anhydride (100.0 g, 1.00 mol) was added. This mixture was stirred for 5 min before 3-mercaptopropionic acid (108.7 g, 1.03 mol) was added, followed by 4-(dimethylamino) pyridine (6.1 g, 0.05 mol). The mixture was stirred overnight at room temperature and then concentrated in vacuo and dissolved in 1 L of ethyl acetate. The obtained solution was acidified with 1 M HCl to pH 1, followed by extraction with ethyl acetate (4 × 300 mL). The extract was dried over anhydrous Na_2_SO_4_, filtered, and concentrated in vacuo. The resulting product was recrystallized from ethyl acetate, giving a white crystalline powder (154.2 g, 75% yield).

^1^H NMR (400 MHz, MeOD, δ) 3.12 (t, *J* = 7.0 Hz, 2H), 2.89 (t, *J* = 6.6 Hz, 2H), 2.68–2.57 (m, 4H) ppm.

^13^C NMR (76 MHz, MeOD, δ): 199.44, 175.60, 175.23, 39.40, 35.12, 29.79, 24.92 ppm.

FTIR (neat, cm^–1^): 1684, 1427, 1404, 1292, 1262, 1191, 1164, 1085, 1002, 971, 909, 797, 752, 657, 627, 484.

### Synthesis of TE-2

To a 2 L three-neck round-bottom flask, equipped with a mechanical stirrer and condenser, 930 mL of toluene was added. Then, TE-1 (83.0 g, 0.40 mol), along with anhydrous sodium sulfate (228.7 g, 1.61 mol) and TsOH (7.7 g, 0.04 mol), were placed in the flask. The mixture was allowed to stir for 10 min before 132 mL of allyl alcohol (112.7 g, 1.61 mol) was added. The mixture was heated to 85 °C and stirred overnight. Subsequently, the mixture was filtered and concentrated in vacuo. The product was purified by means of flash chromatography using neutral alumina as a stationary phase and mixtures of cyclohexane-ethyl acetate as eluent, giving the colorless liquid (101.6 g, 88% yield)

^1^H NMR (400 MHz, CDCl3, δ) 5.98–5.86 (m, 2H), 5.15–5.42 (m, 4H), 4.61 (d, *J* = 5.6 Hz, 4H), 3.16 (t, *J* = 7.0 Hz, 2H), 2.92 (t, *J* = 7.0 Hz, 2H), 2.74–2.64 (m, 4H) ppm.

^13^C NMR (76 MHz, CDCl3, δ) 197.51, 171.63, 171.36, 132.06, 118.62, 118.56, 65.63, 65.58, 60.51, 38.51, 34.47, 29.24, 24.09 ppm.

FTIR (neat, cm^–1^): 1732, 1689, 1648, 1413, 1376, 1343, 1153, 1067, 987, 930.

### Preparation of Resin Formulations

To an amber vial, TMG, PETMP, TE-2, DAA, and TPO-L were added and mixed at RT for 5 min until homogeneity was reached. The resulting solution was bubbled with nitrogen. All resins were prepared with a 100 mol % excess of thiol groups in order to facilitate the thiol-thioester exchange reaction and are summarized in Table 1.

**Table 1:** Compositions of Studied Resins in Mol. Equivalents

	TE-2	DAA	PETMP	TPO-L	TMG
*dyn-1*	1.00	0.00	1.00	0.02	0.03
*dyn-0.75*	0.75	0.25	1.00	0.02	0.03
*dyn-0.5*	0.50	0.50	1.00	0.02	0.03
*dyn-0.25*	0.25	0.75	1.00	0.02	0.03
*dyn-0*	0.00	1.00	1.00	0.02	0.03
*dyn-0.8*	0.80	0.20	1.00	0.02	0.03
*dyn-0.6*	0.60	0.40	1.00	0.02	0.03
*dyn-0.4*	0.40	0.60	1.00	0.02	0.03

### Multimaterial DLP 3D Printing

Multimaterial DLP 3D printing was performed on a dual-valence DLP prototype (W2P Engineering GmbH, Austria). The DLP printer utilized the light engine from In-Vision Digital Imaging Optics GmbH operating at 405 nm with the maximum power of 40 mW/cm^2^ and a pixel size of 50 μm. For creep experiments, strips (40 × 4 × 1 mm) consisting of ten subsequent blocks (4 × 4 × 0.8 mm) were printed using *dyn-1* and *dyn-0.25* formulations. For imprinting, films consisting of two materials were printed (16 mm × 8 mm × 0.8 mm in total, and each block 8 mm × 8 mm × 0.8 mm) using *dyn-1* and *dyn-0.5* formulations. The cube backbone structures were printed by using *dyn-0.75* and *dyn-0* formulations. The drawings of the 3D-printed samples are shown in Figure S1. To improve the visual distinction between two materials, *dyn-1* and *dyn-0.75* formulations were colored using 0.01 wt % Sudan II. All formulations were printed with a layer thickness of 100 μm using a power density of 10 mW/cm^2^ and an exposure time of 240 s (*dyn-1* and *dyn-0.75* resins) and 120 s (*dyn-0.5*, *dyn-0.25*, and *dyn-0* resins) per layer at room temperature. The multimaterial printed objects were rinsed with isopropanol to remove residuals of liquid resin from the surface and postcured upon 405 nm irradiation (Opsytec Dr. Gröbel, 79.3 mW cm^–2^ for 1 min).

Imprinting experiments were performed on the multimaterial samples using stainless steel imprinting stamps. The stamps and samples were heated up to the imprinting temperature in an oven before placing the stamps on the samples for different times. The imprinting experiments were performed at 40, 60, and 80 °C.

### Characterization Methods

^1^H and ^13^C nuclear magnetic resonance spectra (NMR) were recorded on an Avance III 300 MHz spectrometer (Bruker, USA) with deuterated methanol and CDCl_3_ as solvents and TMS as internal standard.

Curing kinetics were followed by FTIR spectroscopy and differential scanning calorimetry (photo-DSC). Photo-DSC measurements were conducted using the Phenyx DSC (Netzsch GmbH, Germany) equipped with an OMNICURE S2000 UV lamp with the light filter, which transmits light in a region between 400 and 500 nm; the overall light power was 15 W cm^–2^. The samples were analyzed in open aluminum crucibles in isothermal mode at 25 °C under a nitrogen flow of 50 mL min^–1^.

FTIR spectra were accumulated utilizing a Vertex 70 spectrometer (Bruker, USA) within 16 scans in transmission mode from 4000 to 900 cm^–1^ with a resolution of 4 cm^–1^, and the absorption peak areas were determined with SpectraGryph software. Ten μL of the resin was placed between two CaF_2_ disks and cured with 405 nm LED light (Opsytec Dr. Gröbel, 2.4 and 9.4 mW cm^–2^) with increasing irradiation times. Evaluation of the curing process was performed by calculating the integral area of the bands in the ranges 2650–2450 cm^–1^ for thiol and 1655–1630 cm^–1^ for C=C, referring to the carbonyl group oscillations at 1740 cm^–1^. To analyze the curing conversion during 3D printing, 5 × 5 × 0.5 mm coupons of each resin material were 3D printed. FTIR spectra were accumulated from two sides of the object by utilizing a Vertex 70 spectrometer (Bruker, USA), averaging 16 scans in ATR mode from 4000 to 400 cm^–1^ with a resolution of 4 cm^–1^. The absorption peak areas were determined with SpectraGryph software. The conversion was calculated using the integral area of the bands in the range 1655–1630 cm^–1^ for C=C, referring to the carbonyl group oscillations at 1740 cm^–1^.

Tensile testing was performed on a Physica MCR501 rheometer (Anton Paar, Austria) with extensional fixture UXF12/CTD at a strain rate of 10% min^–1^ at room temperature. The samples were cured between two glass plates with a 0.1 mm spacer by exposure of the sample to 405 nm LED light (Opsytec Dr. Gröbel, 79.3 mW cm^–2^) for 30 s from each side (total exposure dose is 0.56 J cm^–2^) and cut with a blade into rectangular specimens (0.1 × 5 × 40 mm) for testing. Samples containing Sudan II were cured with a double irradiation dose to ensure complete curing (total exposure dose is 1.12 J cm^–2^). The modulus of elasticity (Young’s modulus) was determined as the slope of the linear fit of the data obtained between 2% to 4% of deformation. The elongation at break was determined as the maximum strain at break. For each experimental condition, 10 specimens were tested in order to perform a statistical analysis of the mechanical testing data.

Relaxation measurements were performed using the Physica MCR501 rheometer (Anton Paar, Austria) with plate–plate geometry and a torque deformation of 3%, under a normal force of 10 N at temperatures between 30 and 80 °C. The specimens were cured upon 405 nm irradiation (Opsytec Dr. Gröbel, 79.3 mW cm^–2^ for 1 min per side (total exposure dose is 1.13 J cm^–2^) in a silicon mold to obtain cylindrical samples with a diameter of 10 mm and height of 1 mm.

The glass transition temperatures were measured with a DSC 4000 instrument (PerkinElmer, USA). All measurements were performed with a heating rate of 10 °C min^–1^ under a nitrogen flow of 50 mL min^–1^ using the samples with a weight of 10 ± 2 mg cured upon 405 nm irradiation (Opsytec Dr. Gröbel, 79.3 mW cm^–2^ for 1 min per side (total exposure dose is 1.13 J cm^–2^). The polymers' *T*_g_ were evaluated in the temperature range from −60 to 50 °C and were determined from the second heating run as an inflection point of the DSC curve. For the statistical evaluation, three samples of each formulation were tested.

All fitting operations were carried out in OriginPro 2024b (64-bit) 10.1.5.132 from OriginLab Corporation (Northampton, USA). The fitting of the stress relaxation data to Maxwell and KWW models was performed using the Levenberg–Marquardt algorithm with the end criteria of tolerance of 1e-9. The fitting of the stress relaxation times to concentration-dependent and concentration-independent Arrhenius–Rouse models was performed using the orthogonal distance regression algorithm with the end criteria of tolerance of 1e-9. The validation of the concentration-dependent Arrhenius-Rouse model was performed using stress relaxation data obtained for formulations *dyn-0.80*, *dyn-0.60*, and *dyn-0.40* using the statistical metrics, such as mean absolute percentage error (MAPE) and predictive coefficient of determination (*R* ^2^_pred_) as validation metrics.

Creep tests were performed on the multimaterial printed samples using the Physica MCR501 rheometer (Anton Paar, Austria) with universal extension geometry (UXF12) with constant extension stress of 50 kPa at 40 °C until 40% of the final deformation. The dimensions of the samples prior to and after the stress relaxation were measured using the stereomicroscope Stemi 2000-C (Zeiss AG, Germany).

The imprints were analyzed by the Stereomicroscope Stemi 2000-C (Zeiss AG, Germany). The surface topography of the imprints was characterized with a confocal microscope MicroProf (Fries Research & Technology GmbH, Germany). The measuring frequency was 320 Hz with a lateral resolution of 20 μm. The 3D and height profiles were extracted from the intensity measurements with the associated software Mark III (Fries Research & Technology GmbH, Germany).

## Results and Discussion

### Curing Kinetics and Material Properties

To study the effect of the dynamic bond concentration on the properties of CANs relying on thiol-thioester exchange reactions, thiol–ene photopolymers were prepared. The allyl monomer, bearing a thioester unit (TE-2), was synthesized according to known methods^65,67^ and incorporated into the resin formulations. Varying the concentration of the dynamic thioester moiety was achieved by subsequently replacing TE-2 with diallyladipate (DAA), an isostructural monomer with an aliphatic backbone (Figure 1a). As a tetrafunctional thiol cross-linker, pentaerythritol tetrakis­(3-mercaptopropionate) (PETMP) was used. Since free -SH groups are required for the thiol-thioester exchange, an excess of 100 mol % of thiol groups was applied in all resin formulations. In order to catalyze the exchange reactions, 3 mol % (with respect to PETMP) of the strong organic base 1,1,3,3,-tetramethylguanidine (TMG) was included. To trigger the thiol–ene click reaction under visible light, 2 mol % (ethyl (2,4,6-trimethylbenzoyl) phenylphosphinate, (TPO-L) was added as a photoinitiator. The final resin formulations are summarized in Table 1.

**Figure 1:**
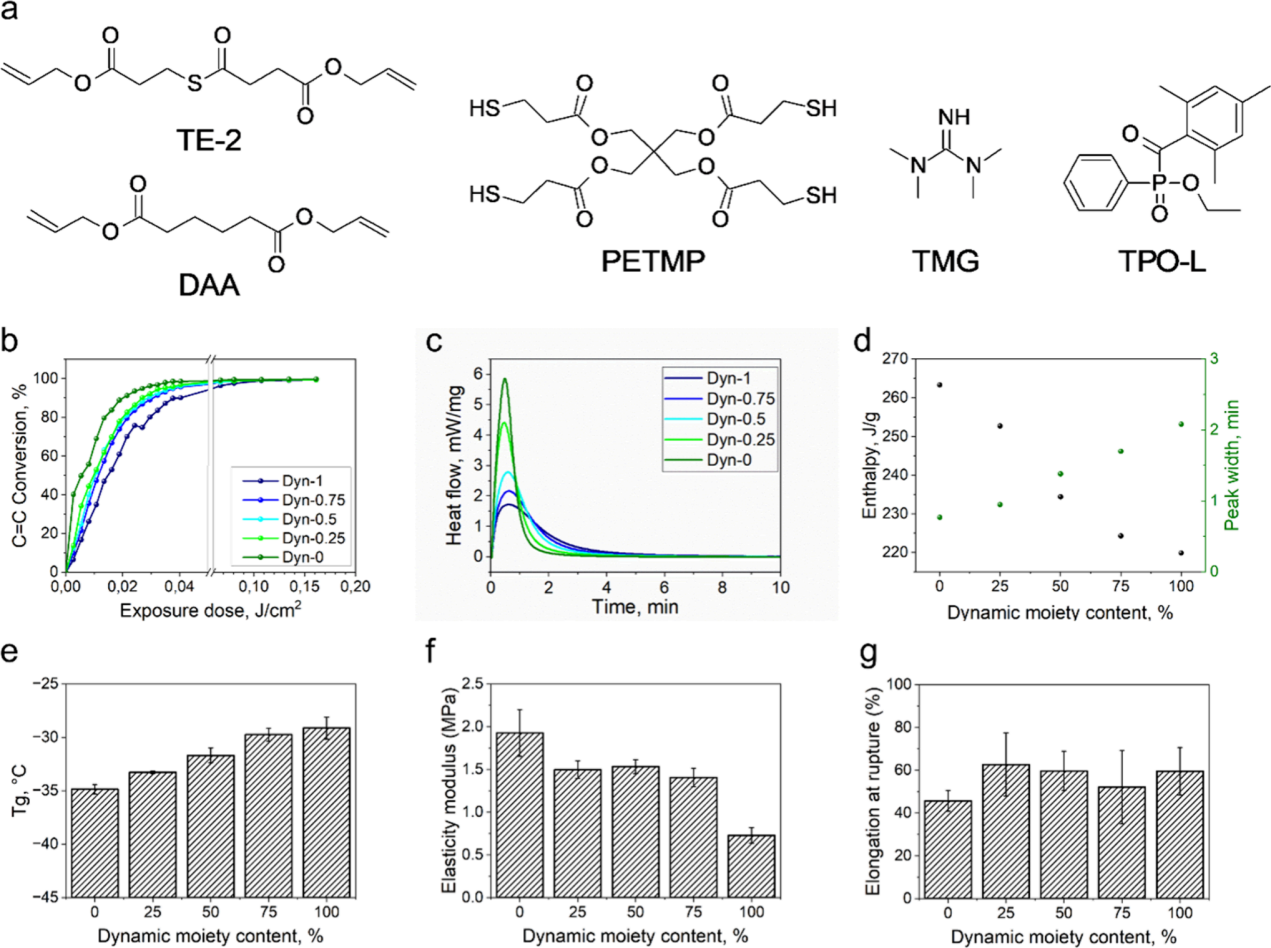
(a) Structures of components; (b) monitoring the conversion of C=C groups in formulations upon light irradiation as obtained from FTIR measurements; (c) monitoring the conversion of the monomers in formulations upon light irradiation as obtained from photo-DSC measurements; (d) curing enthalpy and peak width calculated from the photo-DSC experiments;(e) dependence of glass transition temperatures on the thioester moiety content.

The effect of variation of dynamic bond concentration on the curing kinetics was followed by FTIR spectroscopy. The characteristic thiol (2569 cm^–1^) and alkene (1645 cm^–1^) absorption peaks decreased upon exposure to 405 nm light until full conversion was reached (Figure S2a–e). At an exposure dose of 0.08 J/cm^2^, the alkene conversion exceeded 95% in all formulations tested, indicating a fast cure speed, thus making the materials suitable for 3D printing by digital light processing (DLP). Decreasing the TE-2 concentration led to a slight increase in the C=C conversion rate (Figure 1b). The same phenomenon was observed by using photodifferential scanning calorimetry (photo-DSC) upon 400–500 nm light irradiation (Figure 1c, the raw photo-DSC data are presented on Figure S3). Substitution of the dynamic TE-2 with the nondynamic DAA led to an increase in the polymerization enthalpy and a decay of the peak width, suggesting a faster reaction rate (Figure 1d). It is assumed that the negative effect of TE-2 on the polymerization kinetics is related to the electron-withdrawing effect of the thioester moiety, which reduces the reactivity of the allyl groups in the radical-induced thiol–ene click reaction. Another possible reason might be the high refractive index of the thioester-containing formulations, which can influence light propagation and distribution within the resin.^68^ Higher refractive indices may lead to increased reflection and scattering at interfaces, thereby reducing the light penetration depth and decreasing the effective photocuring rate.

Besides the curing kinetics, the introduction of thioester segments also affected the glass transition point of the cured polymer network. By increasing the amount of TE-2 monomer, the glass transition point also rose linearly from −34.4 to −28.4 °C, which implies the formation of a more rigid network (Figure 1e, the raw DSC scans are presented in Figure S4a–f) and can be an additional reason for the slower curing rates. Although the introduction of TE-2 increased the glass transition temperature, a decrease in Young’s modulus was observed. For the *dyn-0* formulation, the Young’s modulus was found to be 1.93 ± 0.27 MPa, while *dyn-1* showed only 0.73 ± 0.07 MPa (Figure 1f). This can be attributed to a reduction in effective cross-link density and an increase in free volume with the increase in the bulky thioester group, which outweighs the stiffening effect of restricted segmental motion reflected by the higher *T*_g_.^69^ Despite the significant difference in elastic modulus, the elongation at break slightly increased with the increase of TE-2 content, changing from 45.7 ± 4.8 to 59.56 ± 11.1% for *dyn-0* and *dyn-1*, respectively (Figure 1g). The tensile test curves are shown in Figure S5a–f.

### Rheological Investigations

To investigate the effect of dynamic thioester concentration on the exchange reaction kinetics, stress relaxation measurements were performed in a temperature range from 30 to 80 °C on photocured samples. As a reference, the stress relaxation measurement of the *dyn-0* formulation at 80 °C was performed to exclude other factors except the dynamic thiol-thioester exchange (Figure S6). Only certain stress relaxation was observed until reaching the plateau at 52% of the applied stress after 10 min. Continued measurements revealed that the stress dropped to 47% of the initially applied stress after 11 h. This phenomenon could be associated with physical stress relaxation or the thiol-ester exchange reaction. The data obtained were first fitted to the Maxwell model according to the following equation:

$$\frac{G}{G_{0}}=e^{-t/\tau }$$ 1

where *G* describes the shear stress relaxation modulus, *G*_0_ the initial shear stress relaxation modulus (at *t* = 0 s), and τ the stress relaxation time, defined as the time at which the normalized shear modulus reaches 1/e. However, the Maxwell model showed high deviation from the data sets (Figure 2a and Figure S7a–c; Table S1). Decreasing the concentration of TE-2 and lowering the temperature led to a lower degree of conformity. The deviation from the ideal “dynamic behavior” can be related to increasing influence of the diffusion factor, the physical relaxation of internal stresses, and the heterogeneity of the bond exchange reactions.^70^

**Figure 2:**
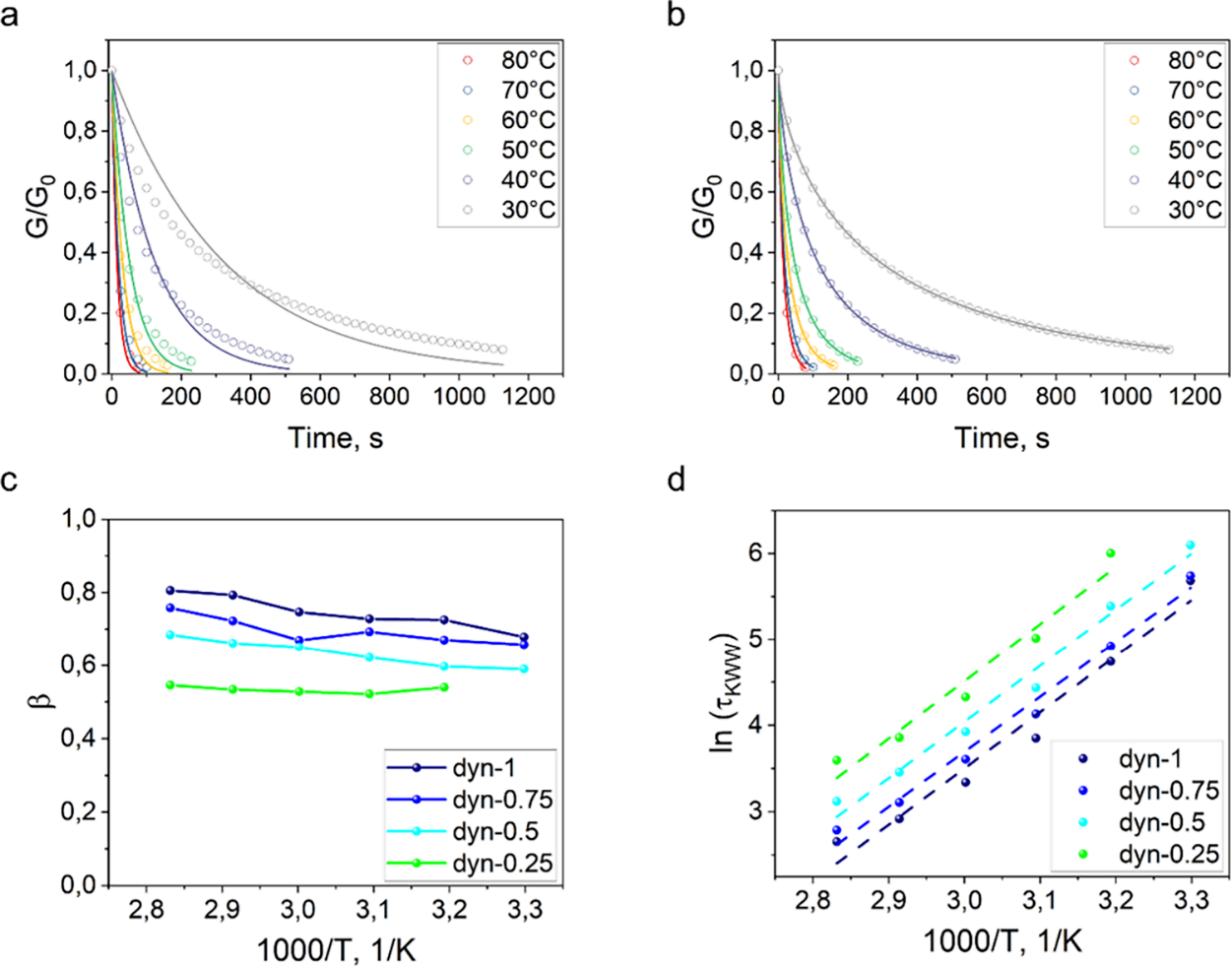
(a) Fitting of the stress relaxation data of *dyn-1* material with the Maxwell model; (b) fitting of the stress relaxation data with the KWW model; (c) dependence of the stretch coefficient on the temperature; (d) Arrhenius plots of the stress relaxation times obtained with the KWW model.

To explore the deviation from the ideal Maxwell behavior the Kohlrausch–Williams–Watts (KWW) stretched exponential equation was used:

$$\frac{G}{G_{0}}=e^{-{\left(\frac{t}{\tau }\right)}^{\beta }}$$ 2

where *G* describes the shear stress relaxation modulus, *G*_0_ the initial shear stress relaxation modulus (at *t* = 0 s), τ the stress relaxation time, and β the stretch coefficient, describing the deviation from classical Maxwell behavior. The updated model showed much better compliance with the experimental data (*R* ^2^ > 0.995, Figure 2b, Figure S7d–f, Table S2). Surprisingly, even for the fully dynamic network, the stretch coefficient was found to be 0.75 ± 0.04, suggesting a nonideal dynamic network (Figure 2c). Lowering the concentration of the thioester monomer led to a reduction of the stretch coefficient, which is associated with an increasing role of nondynamic factors in the stress relaxation.

Additionally, the stress relaxation rate increases with increasing temperature, which serves as a key characteristic of associative CANs. The temperature dependence of the relaxation time can be described according to the Arrhenius law:^14^

$$\tau (T)=\tau_{0}\times e^{-E_{a}/RT}$$ 3

where *E*_a_ is the activation energy of the relaxation process, τ is the relaxation time, *T* the temperature (in K), τ_0_ the characteristic constant, *R* the universal gas constant, equal to 8.314 kJ mol^–1^K^–1^. The stress relaxation times obtained from the KWW model were plotted (Figure 2d) and fitted to eq 3. All Arrhenius approximations showed a similar slope, suggesting the same activation energy for the dynamic exchange reactions. Based on the used models, the activation energy of the dynamic bond exchange reactions was found to be 54.2, 53.0, 54.3, and 55.3 kJ/mol for *dyn-1*, *dyn-0.75*, *dyn-0.5*, and *dyn-0.25,* respectively. However, the obtained data points did not fit the linear Arrhenius model satisfactorily (the *R* ^2^ ranges from 0.94 to 0.98, Figure 2d).

Accordingly, a more refined analysis of the temperature-dependent relaxation behavior was carried out, incorporating the additional relaxation mode attributed to the diffusion process. The role of diffusion was estimated by the Rouse law. The Rouse model describes the conformational dynamics of an ideal chain, where the single-chain diffusion is represented by Brownian motion of beads connected by harmonic springs.^71^ Thus, the relaxation rate, which is defined as the inverse characteristic relaxation time, is represented as the sum of the Arrhenius-driven relaxation rate of the dynamic bond exchange and the Rouse diffusion rate of the reactive sites:^63^

$$\frac{1}{\tau }=k_{\mathrm{obs}}=k_{\mathrm{Arrhenius}}+k_{\mathrm{Rouse}\;\mathrm{diff}}$$ 4

where $k_{\mathrm{Arrhenius}}\approx \exp\left(\frac{E_{a}}{RT}\right)$ , *k*_Rouse diff_ ≈ *T*, τ is the relaxation time, *k*_obs_ is the observed relaxation rate, *k*_Arrhenius_ is the Arrhenius relaxation rate, which is exponentially dependent on the reversed temperature,^14^ and *k*_Rouse diff_ is the Rouse relaxation rate, which is proportional to the temperature.^72^ Combining these two terms for the stress relaxation with inclusion of proportional coefficients allows to get the first approximation of the Arrhenius–Rouse model:

$$\tau =\frac{1}{k_{\mathrm{Arrhenius}}}+\frac{1}{k_{\mathrm{Rouse}\;\mathrm{diff}}}=\frac{1}{A\times \exp\left(-\frac{E_{a}}{RT}\right)}+\frac{1}{B\times T}$$ 5

where *A* and *B* are proportional coefficients for Arrhenius and Rouse modes, respectively. The presented model showed good fitting to the calculated data points with *R* ^2^ > 0.9999 (Figure 3a). The modeling results are presented in Table S3. According to the equations, the Rouse function of the total relaxation time does not change significantly within the measured temperature range. In contrast, the Arrhenius part exhibited a pronounced gradient with changing the temperature. At lower temperatures, the Arrhenius-driven dynamic exchange proceeded very slowly, and the Rouse part played a negligible role in the overall relaxation process. As the temperature increased, the Arrhenius-controlled processes exhibited a pronounced acceleration, whereas the Rouse-related dynamics remained nearly constant, resulting in a notable increase in the relative contribution of diffusion to the overall stress relaxation (Figure 3b).

**Figure 3:**
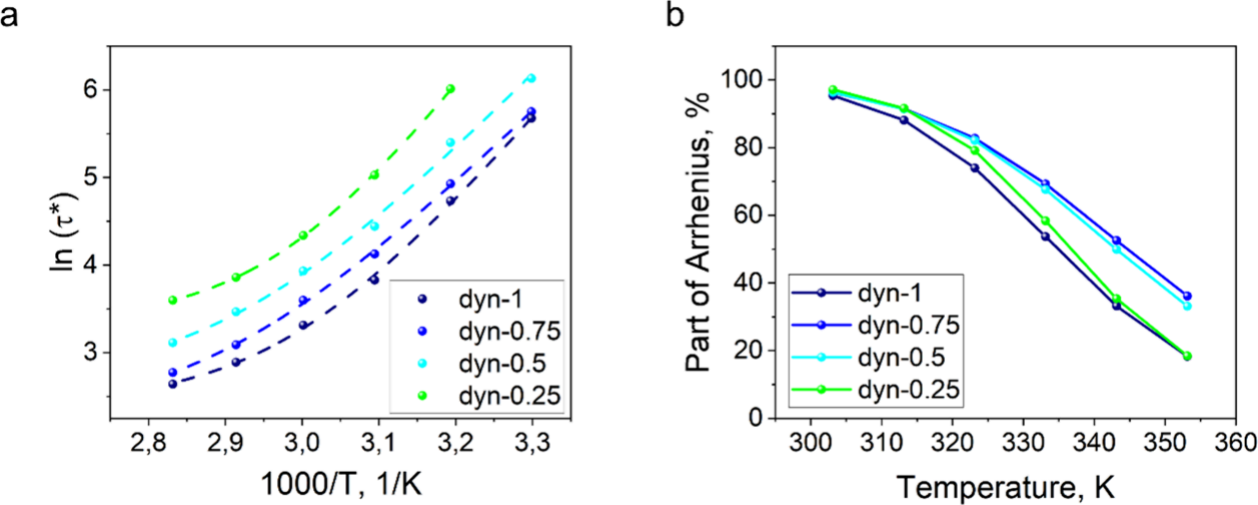
Results of the fitting of the first approximation of the Arrhenius–Rouse model to obtained in KWW stress relaxation times. (a) Fitting stress relaxation times with the first approximation of the Arrhenius–Rouse model; (b) part of the Arrhenius mode in overall relaxation time.

Nonetheless, despite providing a substantially improved fit relative to the Arrhenius model, the obtained model does not adequately reflect the concentration dependence of the stress relaxation time with respect to the dynamic thioester moieties. To demonstrate the dependence of the models on the concentration of the dynamic monomer, both modes were revised. For the Arrhenius mode, the dependence of the stress relaxation rate on the concentration of the dynamic segments remains unclear. It is known from the literature that the activation energy of the exchange reactions does not rely on the concentration of the dynamic species or the catalyst due to the chemical nature of the reaction.^15,37^ On the other hand, stress relaxation experiments have shown that an increase in the concentration of dynamic species leads to an increase in the reaction rate. The thiol-thioester exchange reaction is a second-order reaction,^66^ which allows us to make the assumption that the Arrhenius relaxation rate should be dependent on the concentration in the following way:

$$k_{\mathrm{Arrhenius}}=A_{\mathrm{arr}}\times C^{a_{\mathrm{arr}}}\times \exp\left(-\frac{E_{a}}{RT}\right)$$ 6

where *C* is the concentration of the thioester moiety, *a*_arr_ is the power concentration coefficient, and *A*_arr_ is the Arrhenius distribution coefficient.

For the Rouse mode, it is known that the rate of diffusion is quadratically dependent on the amount of “beads”, or strings, linking the sites in the polymer chain. Hubbard et al.^63^ supposed that the Rouse part depends on the concentration of the catalyst to the power of 2/3. Since the concentration of the dynamic thioester groups could not be represented in the same way, we left the power coefficient as a model parameter:

$$k_{\mathrm{Rouse}\;\mathrm{diff}}=A_{\mathrm{DR}}\times C^{a_{\mathrm{ro}}}\times T$$ 7

where *C* is the concentration of the thioester moiety, *a*_ro_ is the power concentration coefficient, and *A*_DR_ is the Rouse diffusion distribution coefficient. Combining both modes results in the following equation:

$$\tau =\frac{1}{k_{\mathrm{Arrhenius}}}+\frac{1}{k_{\mathrm{Rouse}\;\mathrm{diff}}}=\frac{1}{A_{1}\times C^{k_{1}}\times \exp\left(-\frac{E_{a}}{RT}\right)}+\frac{1}{A_{2}\times C^{k_{2}}\times T}$$ 8

The resulting five-parameter model showed ideal fitting to the experimental data (*R* ^2^ = 1, Figure 4a). The modeling results are presented in Table S4.

**Figure 4:**
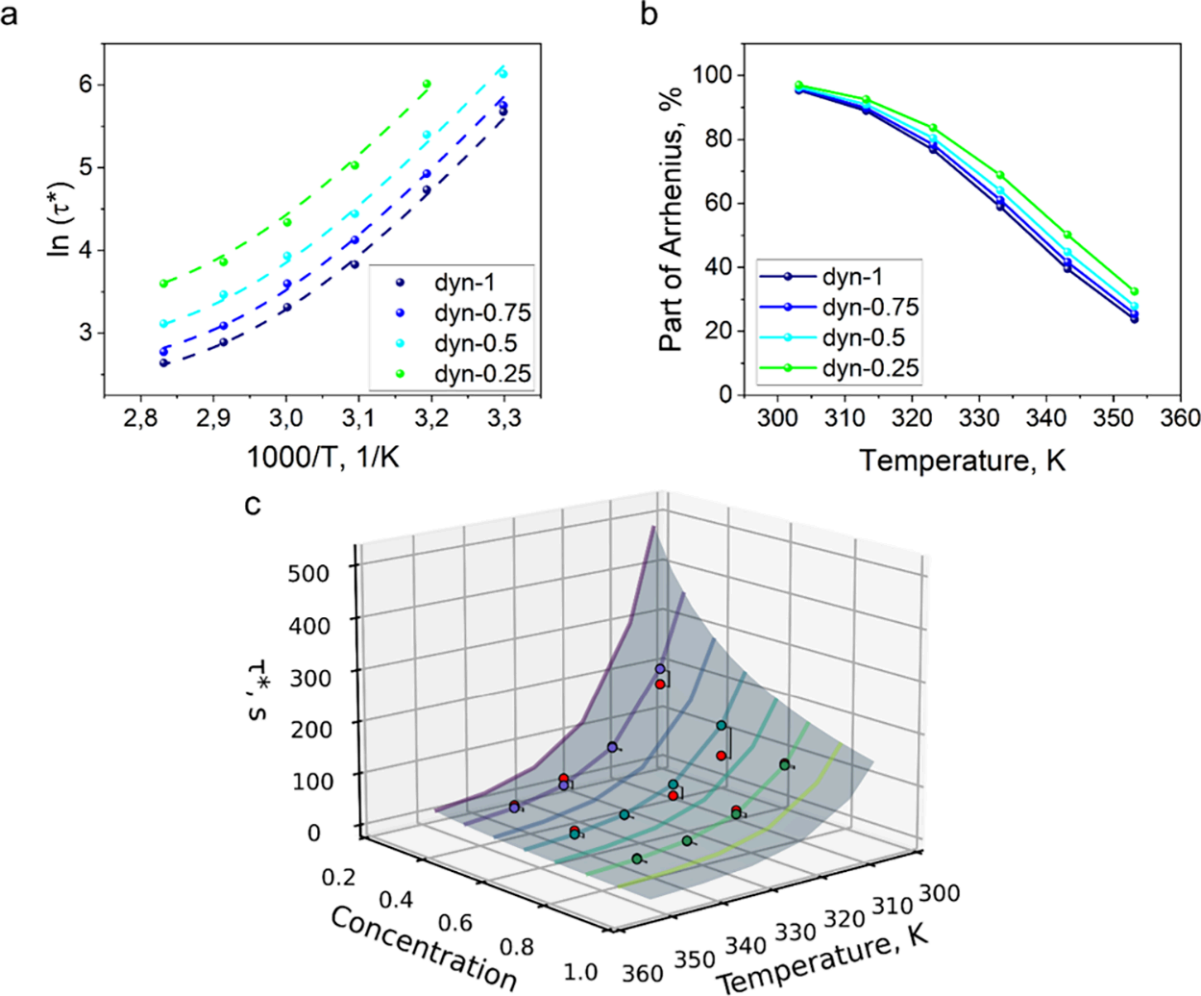
Results of the fitting of the second approximation of the Arrhenius-Rouse model to obtained in KWW stress relaxation times. (a) Fitting of the stress relaxation times with the second approximation of the Arrhenius–Rouse model; (b) part of the Arrhenius mode in overall relaxation time; (c) validation of the stress relaxation times with the second approximation of the Arrhenius–Rouse model–3D plot.

The concentration-dependent model was validated on an independent data set. The stress relaxation measurements of the formulations *dyn-80*, *dyn-60,* and *dyn-40* were performed at 40, 50, 60, and 70 °C and evaluated with the KWW equation in order to obtain the characteristic relaxation times (Figure S8a–c, Table S5). Using the parameters obtained from the fitting model of the original data set, we predicted the relaxation times for the additionally performed measurements. Model performance was evaluated by comparing predicted and observed values using the predictive coefficient of determination (*R* ^2^_p__r__e_ *d*) and the mean absolute percentage error (MAPE), resulting in values of 0.85 and 15.6%, respectively, which indicate a strong predictive capability (Figure 4c).

In both the concentration-dependent and concentration-independent models, the Arrhenius contribution remains predominant at lower temperatures. Upon increasing the temperature, the influence of Rouse-type diffusion becomes progressively more significant in governing the overall stress relaxation behavior (Figure 4b). Surprisingly, the ratio of the Arrhenius and Rouse impacts remains at the same level for all of the studied data sets, which also could be associated with the concentration dependence of the Arrhenius mode.

This comprehensive study of the stress relaxation properties of various dynamic materials, each with distinct dynamic properties, facilitates their targeted selection and blending to meet specific application requirements in DLP 3D printing.

### Multimaterial 3D Printing

To highlight how these differences can be intentionally exploited for designing materials tailored to specific applications, multimaterial 3D printing was performed using a dual-vat DLP 3D printer (Figure 5a). Bottom exposure tests were performed utilizing the *dyn-1* formulation to optimize the printing parameters. The gel formation took place after 20 s of irradiation with 405 nm light (10 mW/cm^2^). However, the green strength of the gel-like sample was not high enough to be removed from the printing platform; thus, prolonged irradiation of 120 s was necessary. The thickness of the printed layer was ∼400 μm, and the obtained irradiation parameters were used for 3D printing with a 100 μm layer thickness. Since the resins were transparent, 0.02 wt % of Sudan II was added as a dye to make the two materials within a single object visually distinguishable. The addition of the dye resulted in slower curing (gel formation occurred after 120 s of irradiation at 10 mW/cm^2^), and the exposure time per layer was therefore increased to 240 s.

**Figure 5:**
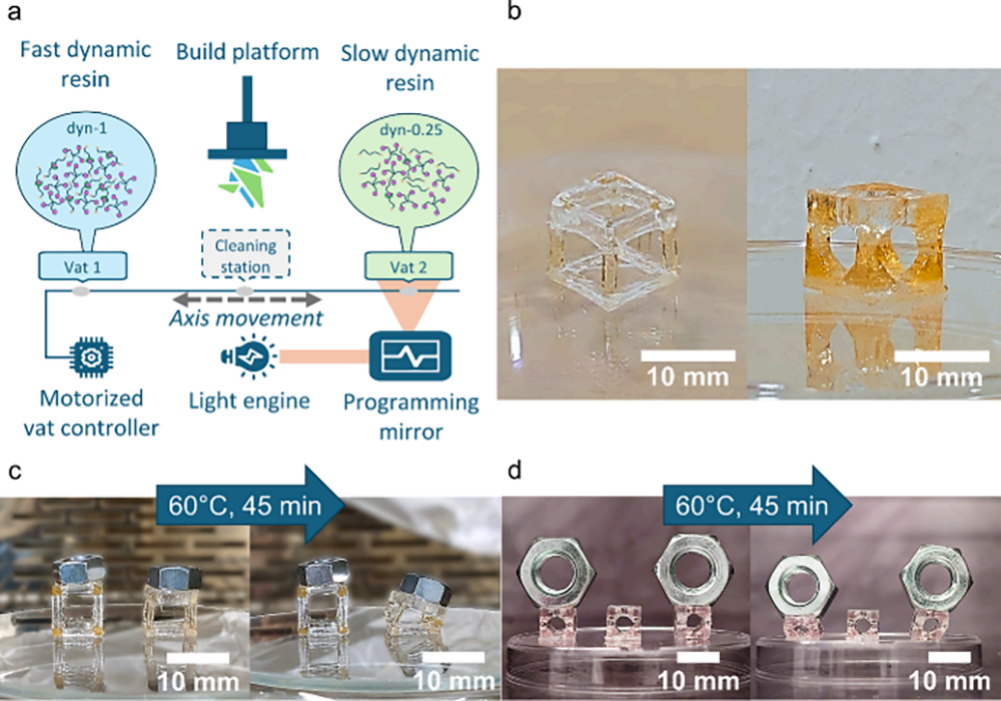
Multimaterial 3D printing. (a) 3D printing setup; (b) 3D-printed cube backbone structures; (c) thermal experiment on the simple cube backbone structure at 60 °C; (d) thermal experiment on the reinforced cube backbone structure at 60 °C.

The addition of 0.02 wt % of Sudan II did not affect the material properties significantly. Stress relaxation experiments of the colored *dyn-1* formulation at 40, 60, and 80 °C showed similar stress relaxation times compared to the noncolored formulation (Figure S9). The obtained data were modeled with the KWW eq (Table S6). However, the stiffness of the colored polymer material was slightly increased, as indicated by a higher elastic modulus (0.98 ± 0.04 MPa for the colored formulation vs 0.73 ± 0.09 MPa for the noncolored one), and the elongation at break decreased (48.6 ± 13.5% for the colored formulation vs 59.6 ± 11.1% for the noncolored one). This behavior could be attributed to higher conversion rates in the Sudan II-containing formulation, resulting from the higher exposure dose used during curing. The tensile test curves of the colored *dyn-1* formulation are shown in Figure S5f.

The determined printing parameters were utilized to print the formulations under investigation. The curing degree of the 3D-printed objects was tested by using FTIR spectroscopy. The conversion of all formulations after curing is presented in Table S7. The achieved degree of curing provided sufficient green strength after printing to allow postcuring without loss of structural integrity. All postcured samples exhibited a conversion of over 95%.

To demonstrate printability, simple and reinforced cube backbones were fabricated using a combination of *dyn-0* and *dyn-0.75* materials (Figure 5b). The bases of the cube backbones were printed with the *dyn-0* formulation, which lacks the dynamic thioester monomer, while the legs were printed with the dynamic *dyn-0.75* material. The incorporation of the dynamic component enables anisotropic structural creep under a vertical load. To demonstrate this behavior, thermal loading of the 3D-printed structures was performed at 60 °C. Vertically aligned dyn-0.75 structures exhibited measurable creep under load, while horizontally aligned ones showed no observable creep, attributable to the permanently cross-linked network of the dyn-0 material (Figure 5c,d).

The different stress relaxation behaviors of the studied materials result in different creep behaviors. To demonstrate it, a consequent pattern of the colored *dyn-*1 material, exhibiting the fastest stress relaxation, and the *dyn-0.25* material, exhibiting the slowest stress relaxation, was printed for the creep test. The obtained samples were subjected to a stress of 50 kPa at 40 °C in the tensile setup until 50% of the final creep deformation was reached. Since the two materials exhibited different creep behavior, differences in the elongation could be observed. The high rate of the dynamic exchange reactions in *dyn-1* material resulted in creep deformation of 77.9%, while *dyn-0.25* showed only 23.6% due to significantly slower exchange dynamics. The samples prior to and after the creep test are shown in Figure 6a,b, respectively.

**Figure 6:**
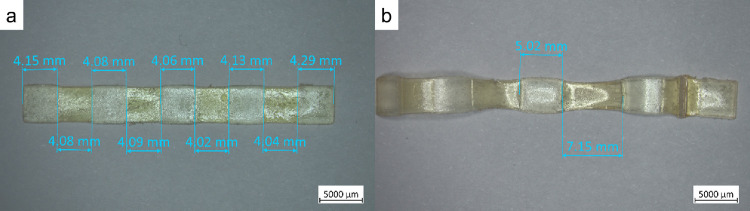
Multimaterial 3D printing: consequent multimaterial samples for the creep test (a) before and (b) after the test.

The creep behavior induced by thiol–thioester exchange reactions enables time- and temperature-controlled thermal imprinting of dynamic polymer materials, whereas permanent networks lacking dynamic bonds exhibit no stress relaxation. Consequently, the required imprinting time and temperature can be tuned by adjusting the concentration of the dynamic moieties. Exploiting the difference in creep performance between two materials within multimaterial systems offers a strategy for evaluating the threshold conditions of time–temperature processing for such objects. Since for most of the applications the time or temperature threshold should be kept narrow, the creep behavior and hence the stress relaxation kinetics of the utilized materials should not differ significantly. Thus, the materials *dyn-1* and *dyn-0.5* were chosen for demonstration due to closer stress relaxation kinetics. The multimaterial samples consisting of two blocks of *dyn-1* and *dyn-0.5* materials were printed and subjected to imprinting utilizing the 3.1 g metal stamp without additional weight at 40, 60, and 80 °C (Figure 7a). Since *dyn-1* is able to release induced stresses much faster (due to a higher thioester content), the imprint was already visible after 15 min at 60 °C, while the imprint on *dyn-0.5* was barely visible only after 40 min at the same conditions; after 80 min, the imprint was sufficiently pronounced (Figure 7c–f). At a temperature of 80 °C, the imprinting of *dyn-1* material became visible after 5 min, while the imprint on *dyn-0.5* material was barely visible after 20 min and a pronounced imprint after 60 min (Figure S10a–d). At 40 °C, the imprinting of *dyn-1* material became visible after 1 h, while the *dyn-0.5* showed barely visible imprint after 6 h (Figure S11a–d). Since each material requires different times to be imprinted, the combination of them sets a threshold between the two times. Thus, this setup could be utilized as a simple time monitoring device, indicating whether the exposure time was within the required range.

**Figure 7:**
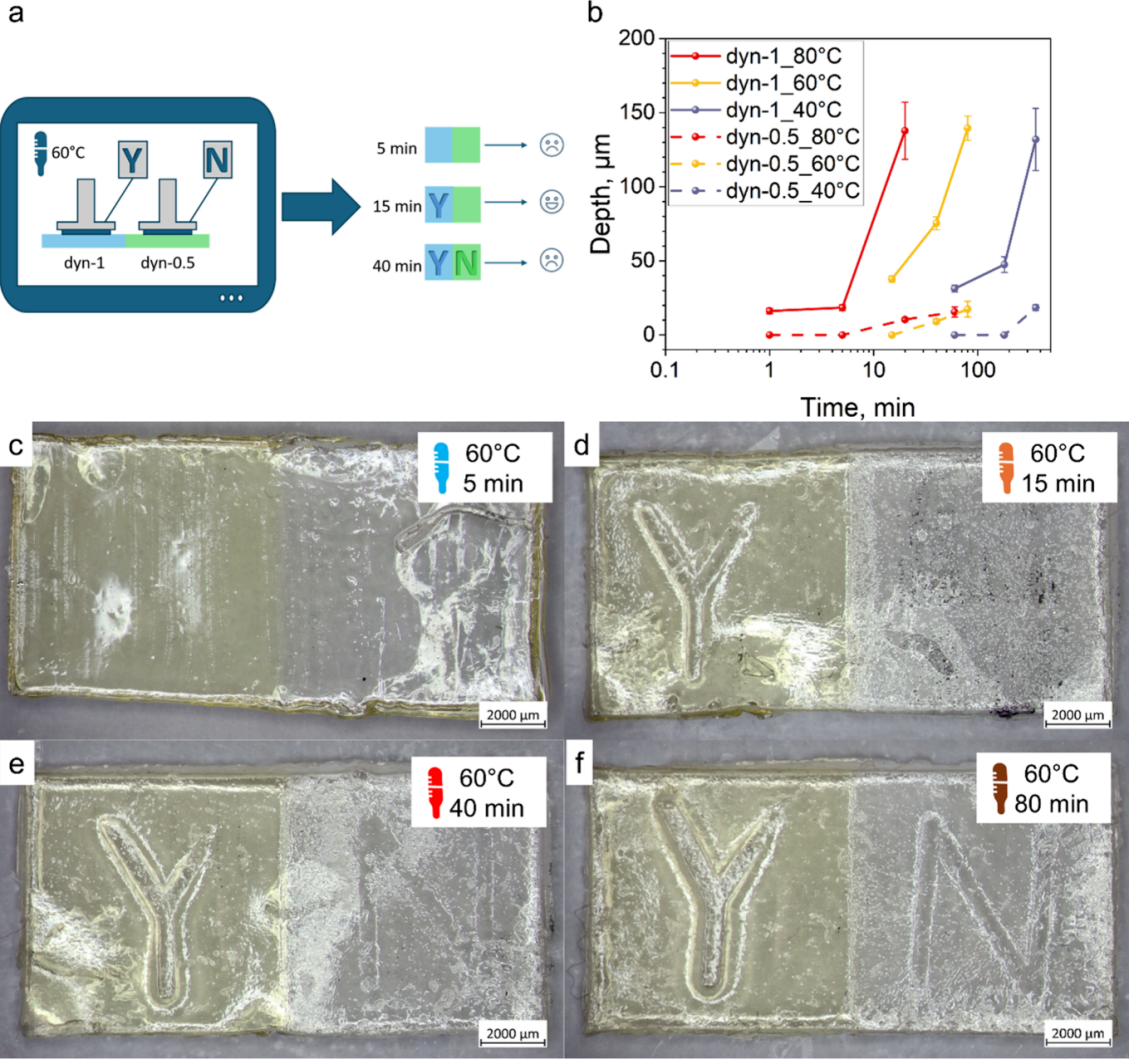
Multimaterial samples after 3D printing for imprint testing. (a) imprinting experiment setup; (b) dependence of imprinting depth on the imprinting duration at different temperatures for materials *dyn-1* and *dyn-0.5*; microscope pictures of the samples imprinted at 60 °C after: (c) 5 min of the imprinting; (d) 15 min of the imprinting; (e) 40 min of the imprinting; (f) 80 min of the imprinting.

However, the visibility of imprinting cannot entirely characterize the imprinting process. A better representation of the imprinting process is the imprinting depth, which is defined by the creep deformation of the sample. The surface topology scans showed the dependence of the imprinting depth on the nature of the material, temperature, and time of the imprinting (Figure 7b). After 40 min of imprinting at 60 °C, the *dyn-1* material exhibited an imprint depth of 75.4 ± 4.4 μm, whereas the *dyn-0.5* material reached only 9.1 ± 1.7 μm, resulting in a barely visible imprint. After 80 min, the imprint became visible and the pattern depth reached 17.4 ± 5.3 μm, while *dyn-1* showed a pattern depth of 139.5 ± 8.2 μm. The same effect was found at 80 °C: the imprinting depth of *dyn-0.5* was significantly less than that for *dyn-1*. In contrast, at 40 °C, the *dyn-0.5* material exhibited almost no measurable imprint depth; the pattern became visually detectable after 6 h of imprinting. The 3D surface topographies of the resulting imprints at 40, 60, and 80 °C are presented in Figures S12 and S13.

Given that imprinting is governed by the creep response of the material, the duration of the process can be modulated through appropriate selection of the stamp’s weight and cross-sectional area, thereby providing enhanced control over imprinting times to target various application demands.

## Conclusions

The variation of dynamic cross-links was utilized to tune stress relaxation kinetics of thiol-thioester CANs. The investigation of the exchange reaction kinetics was performed using different models. The fast rate of the thiol-thioester exchange reaction resulted in a significant deviation from the typical Maxwell behavior. The universal stretched exponential model showed significantly better fitting of the data sets according to the KWW equation. The further analysis of the obtained parameters showed increasing deviation from ideal dynamic behavior when decreasing the concentration of dynamic cross-links, highlighting the growing diffusion factor. To evaluate the role of diffusion, the Arrhenius–Rouse model was applied in both concentration-dependent and concentration-independent forms. The implemented model demonstrated excellent agreement with the experimental data, yielding an *R* ^2^ value of 1. The model was validated using an independent data set comprising 12 stress relaxation data points from the *dyn-0.80*, *dyn-0.60*, and *dyn-0.40* formulations, resulting in a predictive coefficient of determination (*R* ^2^_p__r__e_ *d*) of 0.85. The influence of diffusion on the stress relaxation kinetics was found to increase with temperature, corresponding to the acceleration of Arrhenius-type dynamic exchange reactions. Furthermore, a decrease in the concentration of thioester groups resulted in an enhanced contribution of diffusion, in good agreement with the model predictions. This model enables the prediction of stress relaxation behavior as a function of the thioester group concentration within the polymer network.

Materials with distinct stress relaxation behaviors were processed using a dual-vat, multimaterial 3D printer. The resulting multimaterial samples exhibited enhanced creep performance, attributed to the pronounced differences in the stress relaxation kinetics of the constituent materials. Under tensile creep testing at 40 °C and 50 kPa, the *dyn-1* domains showed an elongation of 77.9%, while the *dyn-0.25* domains exhibited only a 23.6% deformation. Moreover, the differential creep behavior within the multimaterial architecture was exploited for a time–temperature sensing application utilizing thermal imprinting. Such samples exhibit strong potential for monitoring storage and curing conditions through their creep-dependent imprint response.

## Supplementary Material



## Notes

Supporting Information contains the plots of raw experimental data, calculation resulting tables, microscopy pictures, and topology scans of thermal imprinting experiments (PDF)
