# Supplementary material for: Multimaterial Digital Light Processing Three-Dimensional Printing of Materials with Different Relaxation Kinetics
Source: ACS Appl Polym Mater. 2025 Oct 30;7(21):14581–92. doi: 10.1021/acsapm.5c02906 (PMC12626240; doi:10.1021/acsapm.5c02906)
Supplement: Supplementary file 1 [file ap5c02906_si_001.pdf]

Supporting Information

Multi-Material DLP-3D Printing of Materials with Different Relaxation Kinetics

Roman Korotkov[1], Milena Gleirscher[1], Sandra Schlögl[1] and Elisabeth Rossegger[1,2*]*

[1] R. Korotkov, M. Gleirscher, E. Rossegger, S. Schlögl - Polymer Competence Center Leoben GmbH, Sauraugasse 1, Leoben, 8700, Austria

E-mail: elisabeth.rossegger@pccl.at, sandra.schloegl@pccl.at

[2] E. Rossegger – Institute for Chemistry and Technology of Materials, Graz University of Technology, Stremayrgasse 9/V, Graz, 8010, Austria

Results

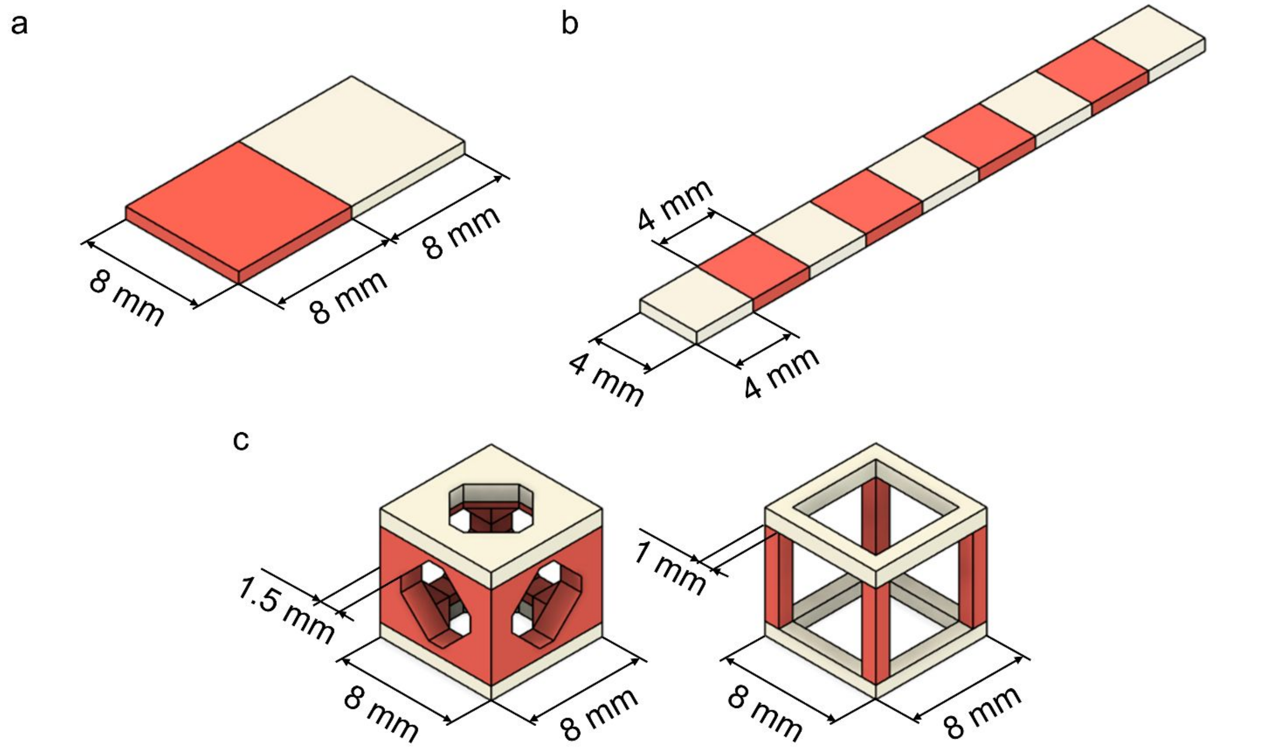

Figure S1. Sketches of 3D printed samples utilized for (a) thermal imprinting; (b) creep tests; (c) .

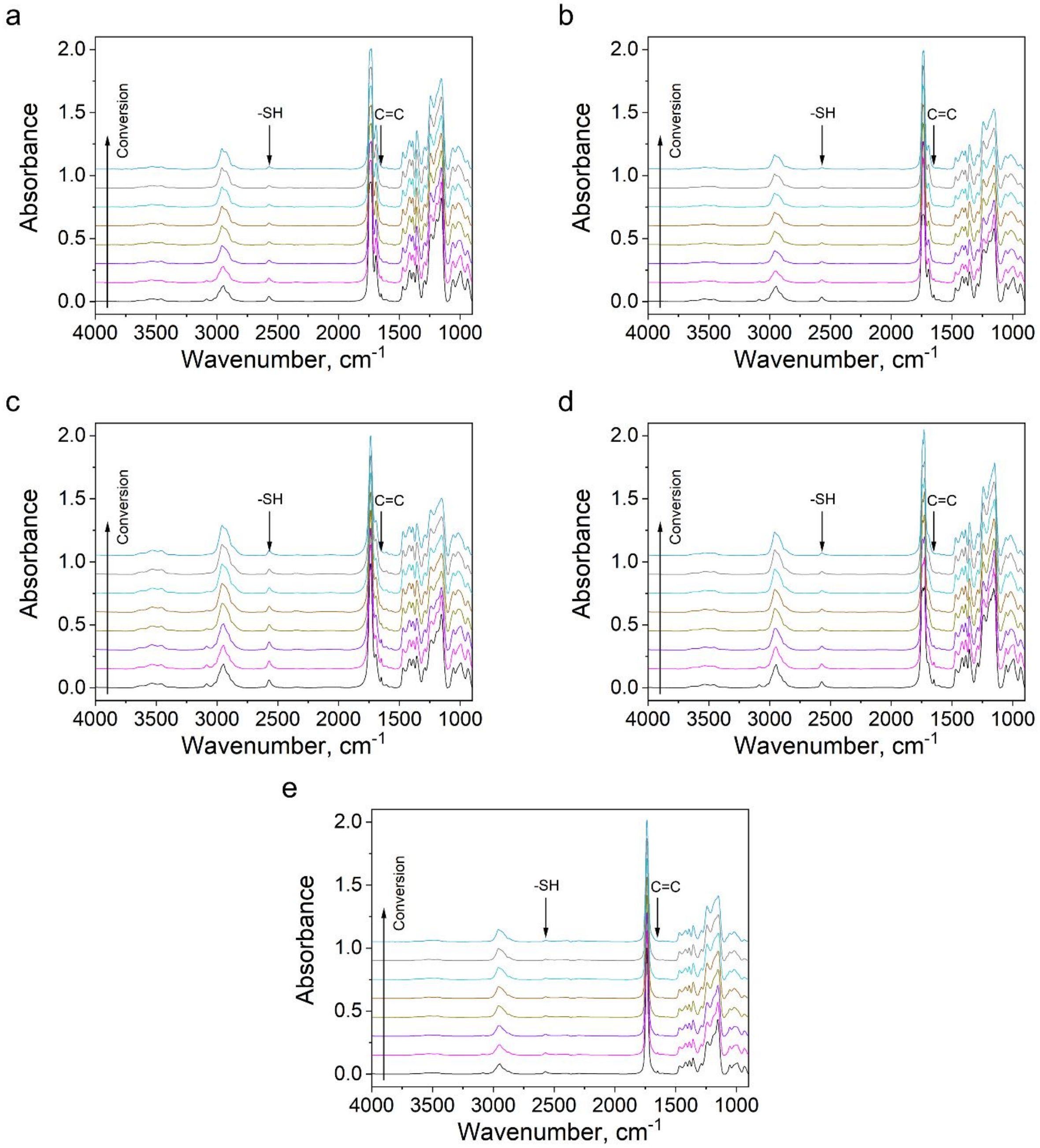

Figure S2. Raw FTIR data of the curing kinetics measurements of formulations: (a) dyn-1; (b) dyn-0.75; (c) dyn-0.5; (d) dyn-0.25; (e) dyn-0.

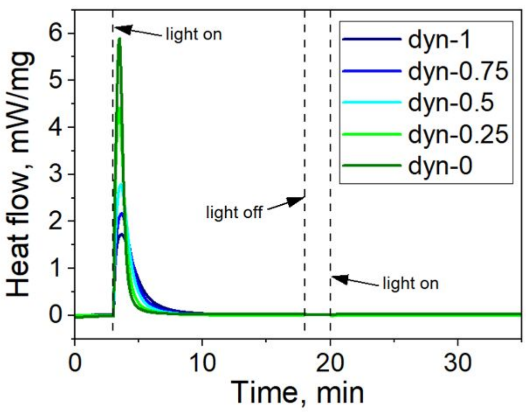

Figure S3. Raw photo-DSC data of the curing kinetics measurements of the studied formulations.

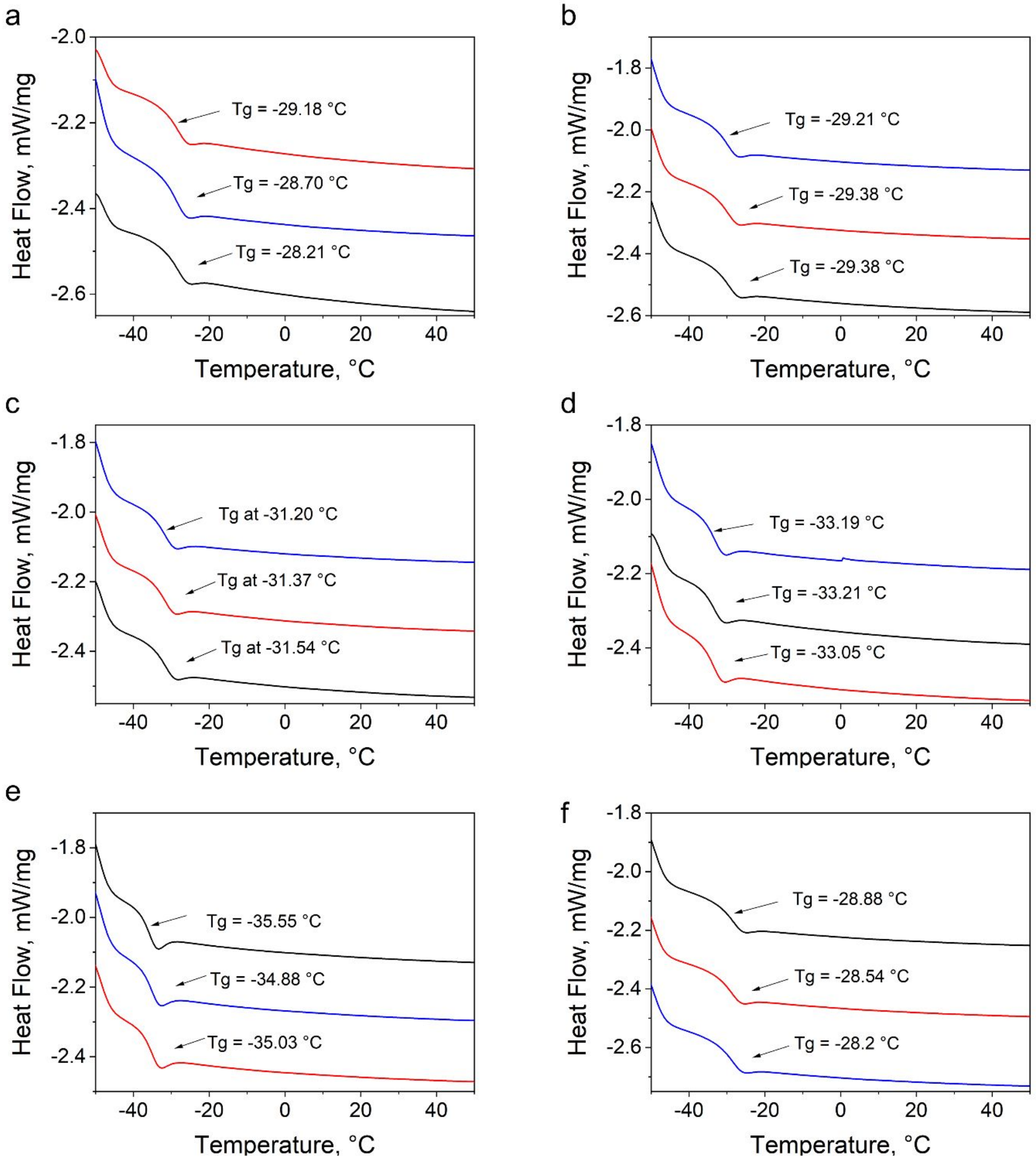

Figure S4. Raw DSC data of the cured formulations: (a) dyn-1; (b) dyn-0.75; (c) dyn-0.5; (d) dyn-0.25; (e) dyn-0; (f) dyn-1+Sudan II.

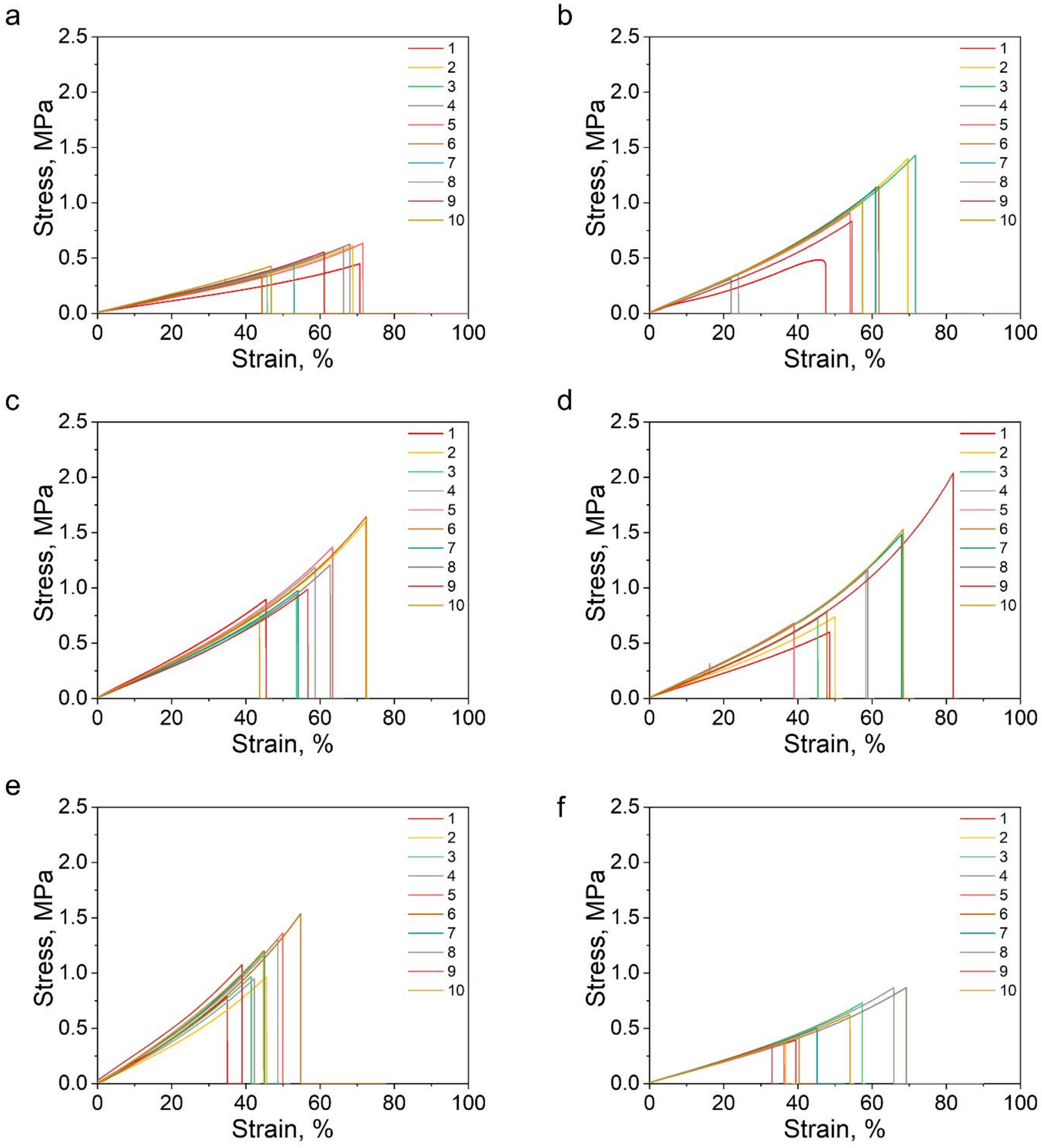

Figure S5. Tensile test curves of the formulations: (a) dyn-0; (b) dyn-0.25; (c) dyn-0.5; (d) dyn-0.75; (e) dyn-1; (f) dyn-1 + Sudan II.

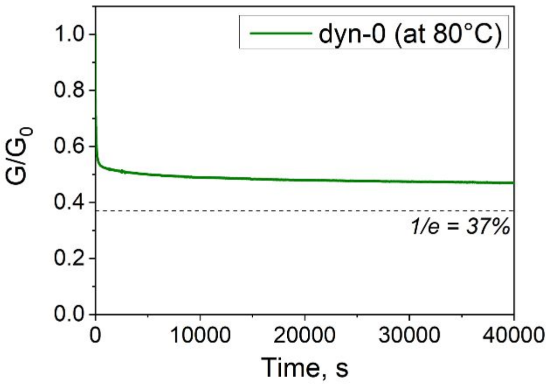

Figure S6. Stress relaxation data of the reference formulation dyn-0 measured at 80°C.

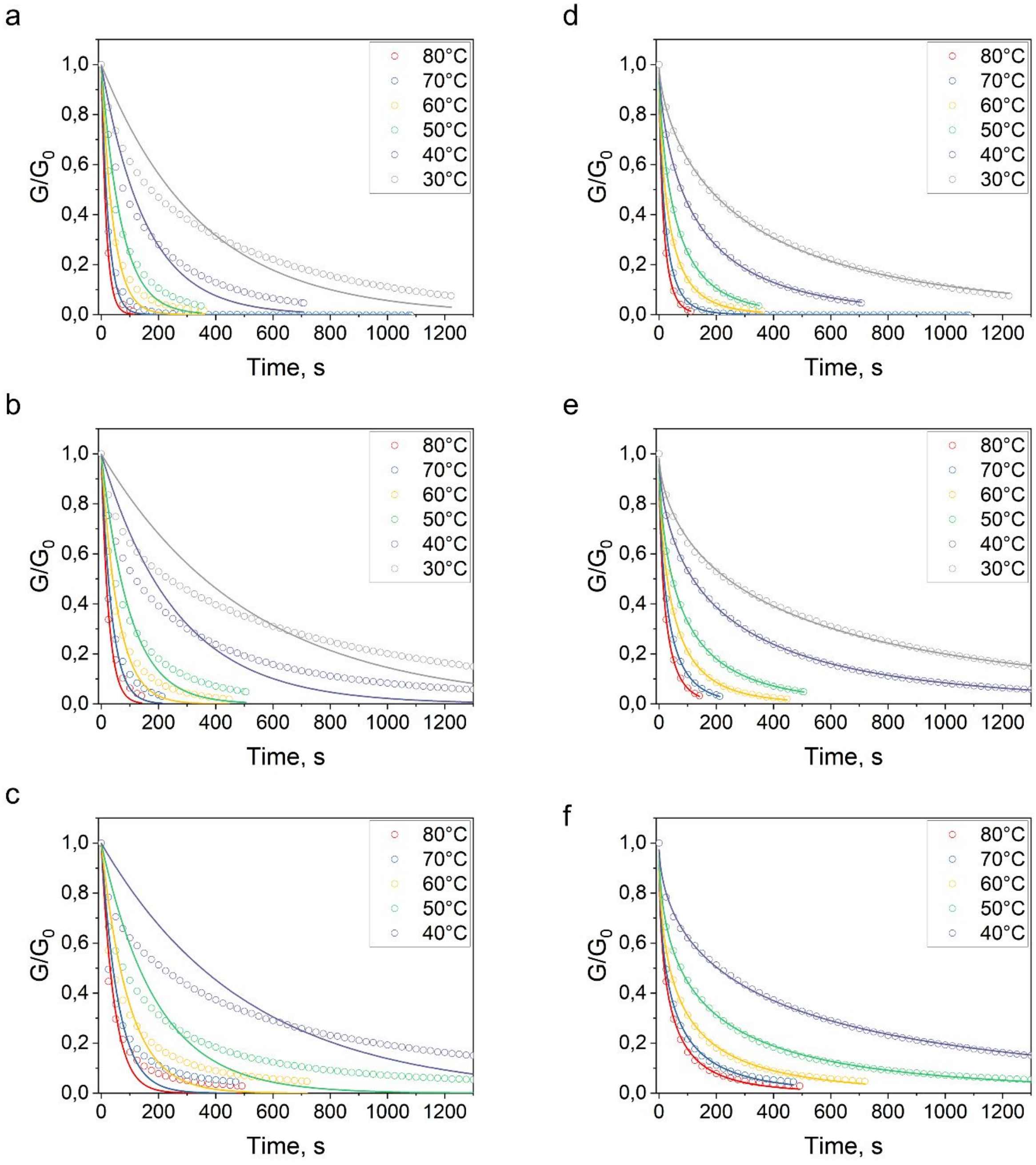

Figure S7. Modelling of the stress relaxation data with: the simple Maxwell model: (a) dyn-0.75; (b) dyn-0.5; (c) dyn-0.25; KWW model: (d) dyn-0.75; (e) dyn-0.5; (f) dyn-0.25.

Table S1. Results of the modelling of the stress relaxation data with the simple Maxwell model.

Formulation	T, °C	Tau, s		Statistics	
		Value	Standard Error	Reduced Chi-Sqr	Adj. R-Square
dyn-1	80	15,00049	0,17457	0,00101	0,9782
	70	19,66102	0,21281	0,00115	0,9752
	60	30,58179	0,32775	0,00175	0,95924
	50	51,33413	0,4856	0,00228	0,94749
	40	124,95067	0,77888	0,00239	0,94527
	30	323,90802	1,56325	0,00365	0,90918
dyn-0.75	80	17,57194	0,21943	0,00137	0,96617
	70	24,62735	0,11607	2,73E-04	0,96989
	60	41,62858	0,41523	0,00207	0,93221
	50	69,65898	0,62433	0,00278	0,92952
	40	155,20826	1,04448	0,00349	0,90955
	30	348,01188	1,74437	0,00423	0,89003
dyn-0.5	80	25,38814	0,3762	0,00278	0,92563
	70	36,20094	0,47807	0,00315	0,91068
	60	58,29164	0,55608	0,00265	0,91518
	50	98,86426	0,94548	0,0045	0,86705
	40	262,10532	1,54651	0,00455	0,8497
	30	519,21645	2,62369	0,00643	0,80395
dyn-0.25	80	44,76239	0,5814	0,00377	0,823
	70	59,24865	0,8108	0,00553	0,76841
	60	95,83866	1,0726	0,00599	0,75051
	50	192,25508	1,58904	0,00655	0,72843
	40	505,59018	2,73086	0,00734	0,73318

Table S2. Results of the modelling of the stress relaxation data with the KWW model.

Formulation	T, °C	tau, s		beta, [1]		Statistics	
		Value	Standard Error	Value	Standard Error	Reduced Chi-Sqr	Adj. R-Square
dyn-1	80	14,1869	0,06103	0,80533	0,00427	8,75E-05	0,99811
	70	18,49546	0,05909	0,79318	0,0031	6,17E-05	0,99867
	60	28,20391	0,06856	0,74641	0,0021	5,02E-05	0,99883
	50	46,99692	0,07661	0,72768	0,00141	3,63E-05	0,99916
	40	114,3767	0,07152	0,72537	5,53E-04	1,29E-05	0,9997
	30	293,726	0,08917	0,67789	2,58E-04	7,10E-06	0,99982
dyn-0.75	80	16,24379	0,05527	0,75795	0,00294	5,78E-05	0,99857
	70	22,37232	0,02398	0,72211	8,09E-04	7,40E-06	0,99918
	60	36,85549	0,07805	0,669	0,00138	4,28E-05	0,9986
	50	62,24374	0,07083	0,69233	8,70E-04	2,19E-05	0,99945
	40	137,0456	0,10037	0,66944	5,45E-04	1,90E-05	0,99951
	30	311,0316	0,22001	0,6574	5,71E-04	3,91E-05	0,99898
dyn-0.5	80	22,60169	0,06194	0,68387	0,002	4,53E-05	0,99879
	70	31,69436	0,06053	0,66064	0,00129	2,94E-05	0,99917
	60	50,67671	0,05712	0,65055	7,04E-04	1,60E-05	0,99949
	50	84,3695	0,08471	0,62156	6,35E-04	1,98E-05	0,99941
	40	218,8022	0,10866	0,59665	2,76E-04	1,19E-05	0,99961
	30	445,3524	0,30969	0,5896	4,74E-04	4,63E-05	0,99859
dyn-0.25	80	36,45544	0,13845	0,54548	0,0016	1,02E-04	0,9952
	70	47,32204	0,13166	0,53372	0,00119	6,85E-05	0,99713
	60	75,63986	0,12885	0,52799	7,26E-04	4,03E-05	0,99832
	50	150,2735	0,15719	0,5214	4,44E-04	2,96E-05	0,99877
	40	404,0851	0,184	0,53974	2,30E-04	1,59E-05	0,99942

Table S3. Results of the modelling of the stress relaxation data with the simple A-R model.

Formulation		dyn-0.25	dyn-0.5	dyn-0.75	dyn-1
A	Value	11067,99	8809,5737	8469,768	10007,551
	Standard Error	218,54921	603,35372	295,47116	409,8336
B	Value	10524,645	5385,8085	3613,2108	4102,9742
	Standard Error	216,56107	883,81219	357,73353	289,3252
c	Value	1,649E-13	1,107E-10	2,223E-10	1,283E-12
	Standard Error	1,123E-13	2,131E-10	2,104E-10	1,682E-12
Statistics	Reduced Chi-Sqr	0,05979	1,25989	0,26885	0,36839
	Adj. R-Square	1	0,99996	0,99998	0,99997

Table S4. Results of the modelling of the stress relaxation data with the concentration dependent A-R model.

A1	Value	9,08E+10
	Standard Error	7,47E+10
k1	Value	0,93687
	Standard Error	0,08155
Ea	Value	77554,14
	Standard Error	2128,702
A2	Value	2,70E-04
	Standard Error	1,57E-05
k2	Value	0,62338
	Standard Error	0,11687
Statistics	Reduced Chi-Sqr	0,00265
	Adj. R-Square	1

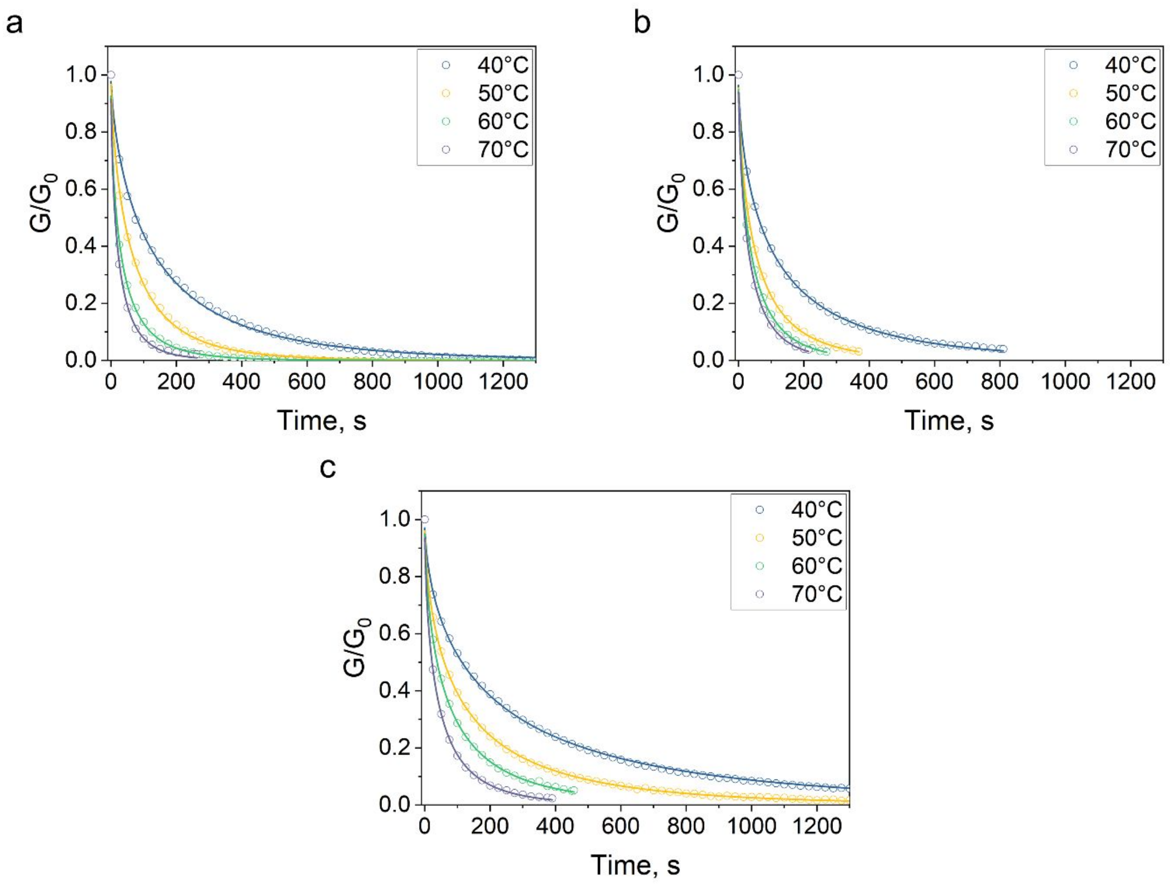

Figure S8. Modelling of the stress relaxation data for validation of the concentration-dependent Arrhenius-Rouse model with the KWW model: (a) dyn-0.8; (b) dyn-0.6; (c) dyn-0.4.

Table S5. Results of the modelling of of the stress relaxation data for validation of the concentration-dependent Arrhenius-Rouse model with the KWW model.

Formulation	T, °C	tau, s		beta, [1]		Statistics	
		Value	Standard Error	Value	Standard Error	Reduced Chi-Sqr	Adj. R-Square
dyn-0.80	40	0.67481	9.18486E-4	134.63259	0.19014	7.05136E-5	0.99694
	50	0.69592	0.00162	67.06994	0.15611	9.95363E-5	0.99416
	60	0.61987	8.25347E-4	31.6261	0.04869	1.75211E-5	0.99763
	70	0.63247	0.00251	22.80295	0.09958	1.04167E-4	0.99605
dyn-0.60	40	0.61359	4.99339E-4	110.64673	0.09454	1.84726E-5	0.99941
	50	0.65698	7.92208E-4	54.79628	0.06484	1.93461E-5	0.99945
	60	0.66591	0.00108	40.28149	0.06331	2.557E-5	0.99928
	70	0.66387	0.00155	33.05572	0.07466	4.31377E-5	0.9988
dyn-0.40	40	0.5787	2.32119E-4	214.75496	0.10884	1.17413E-5	0.99912
	50	0.59157	2.78933E-4	111.76038	0.06427	8.09398E-6	0.99957
	60	0.60139	7.34861E-4	70.01384	0.08798	2.45462E-5	0.99922
	70	0.61451	9.79795E-4	40.83304	0.07215	2.92712E-5	0.99897

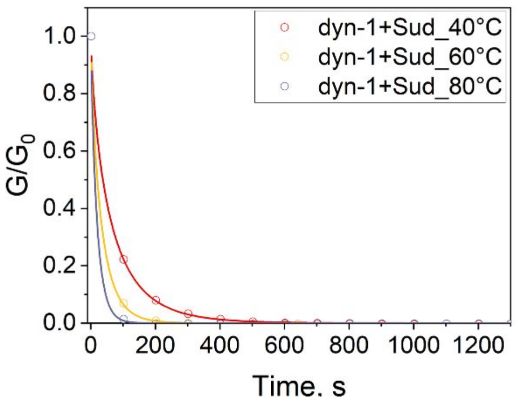

Figure S9. Modelling of the stress relaxation data of the colored with Sudan II dyn-1 formulation measured at 40°C, 60°C and 80°C with the KWW model.

Table S6. Results of the modelling of the stress relaxation data of the colored with Sudan II dyn-1 formulation with the KWW model.

Formulation	T, °C	tau, s		beta, [1]		Statistics	
		Value	Standard Error	Value	Standard Error	Reduced Chi-Sqr	Adj. R-Square
dyn-0.80	40	0.7762	0.00187	60.15709	0.12752	2.14535E-5	0.9981
	60	0.85102	0.00469	31.18201	0.13616	5.32561E-5	0.99735
	80	0.91875	0.00594	18.7041	0.088	4.08857E-5	0.99358

Table S7. Conversion of the resins after 3D printing.

	C=C conversion, %		
Formulation	bottom	top	average
dyn-0	96.4	88.3	92.3
dyn-0.25	98.4	95.0	96.7
dyn-0.5	97.8	60.8	79.3
dyn-0.75	97.2	61.2	79.2
dyn-1	87.1	89.5	88.3
dyn-1 + Sudan II	84.5	63.4	74.0

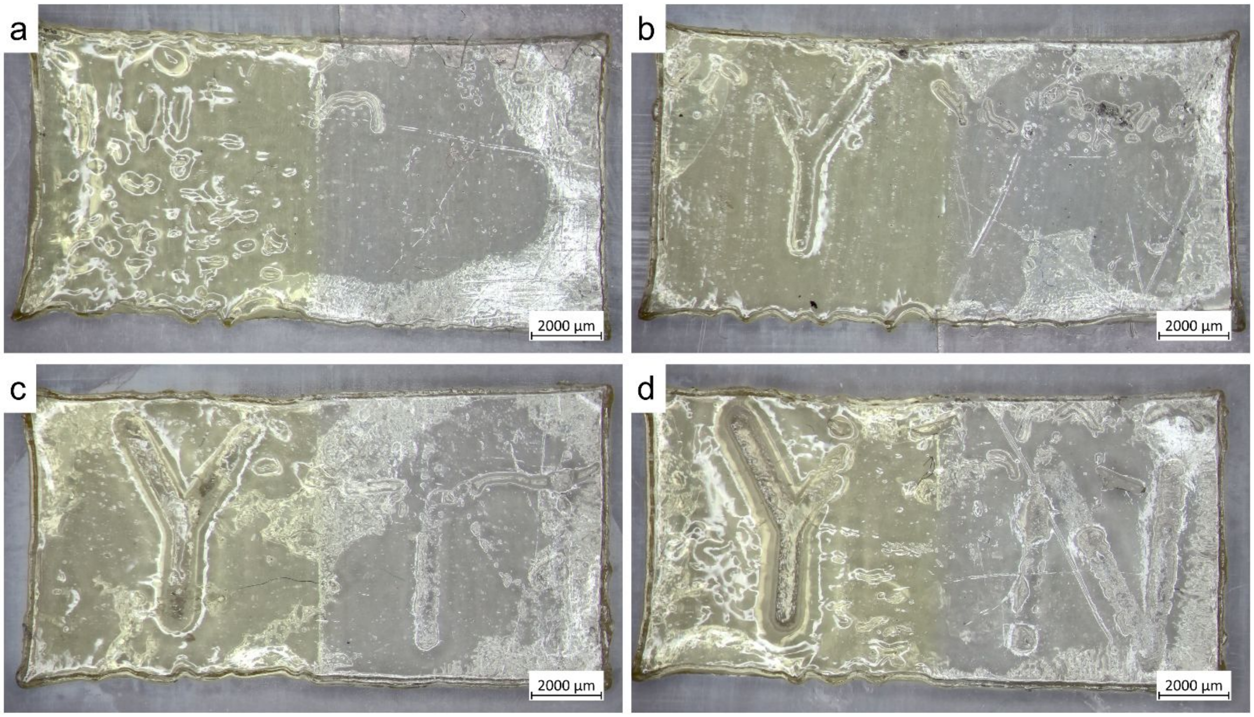

Figure S10. Microscope pictures of the samples imprinted at 80 °C after: (a) 1 minute of the imprinting; (b) 5 minutes of the imprinting; (c) 15 minutes of the imprinting; (d) 40 minutes of the imprinting.

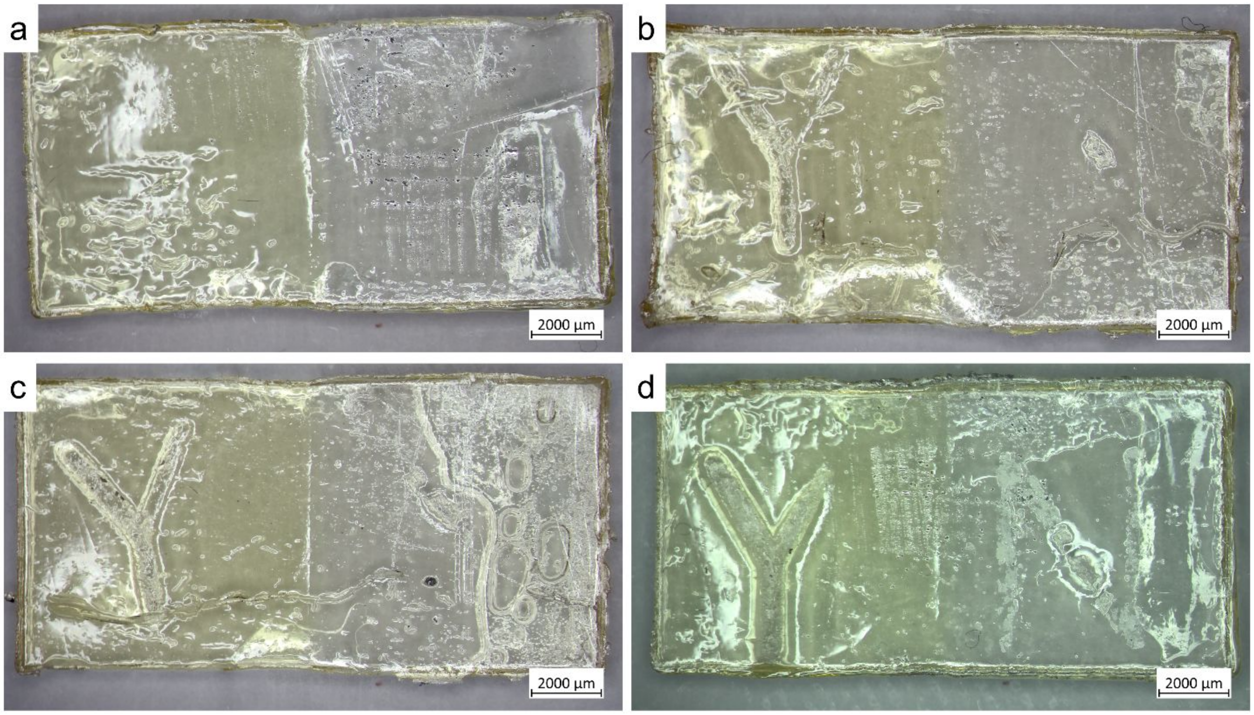

Figure S11. Microscope pictures of the samples imprinted at 40 °C: (a) 30 minutes of the imprinting; (b) 1 hour of the imprinting; (c) 3 hours of the imprinting; (d) 6 hours of the imprinting.

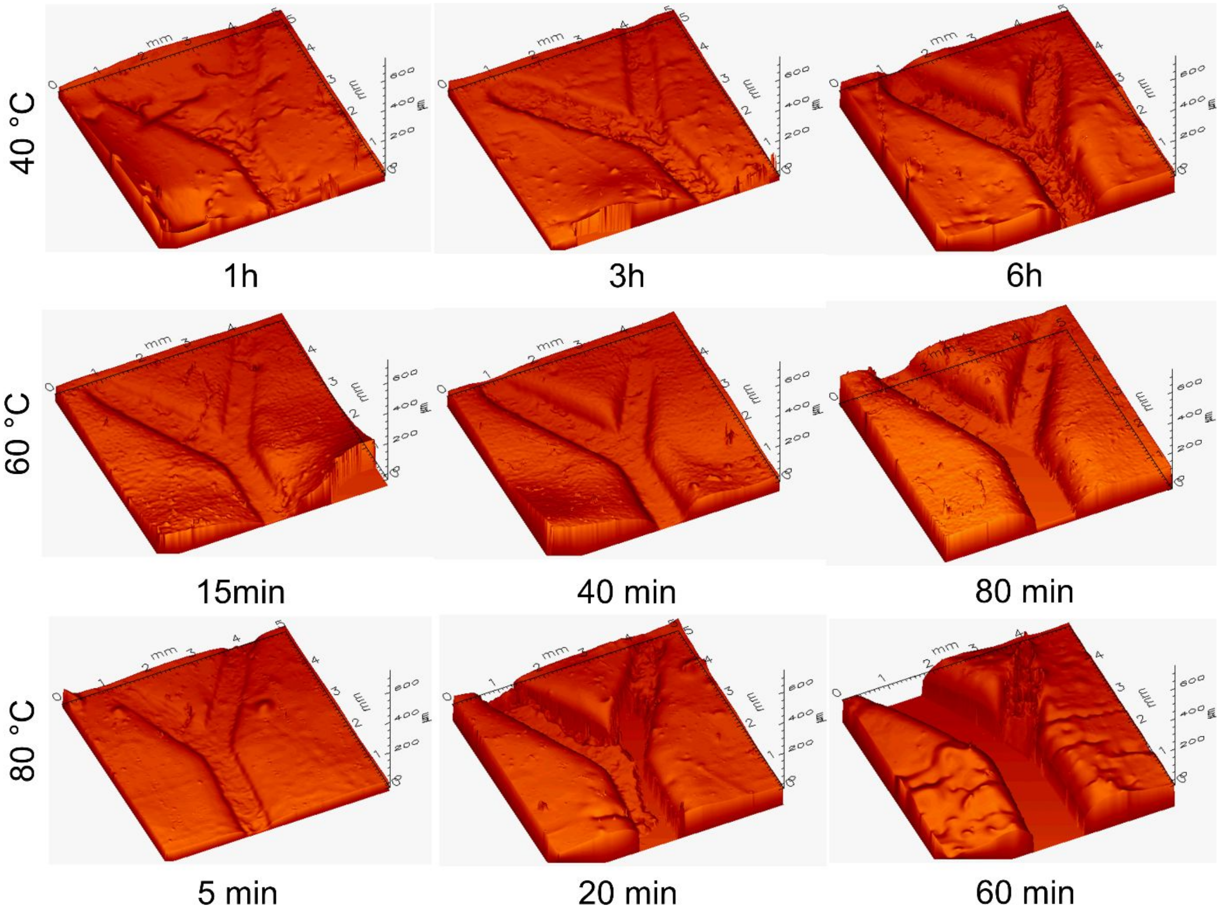

Figure S12. 3D plots of topology surface scans of the *dyn-1* material imprinted at different temperatures and times.

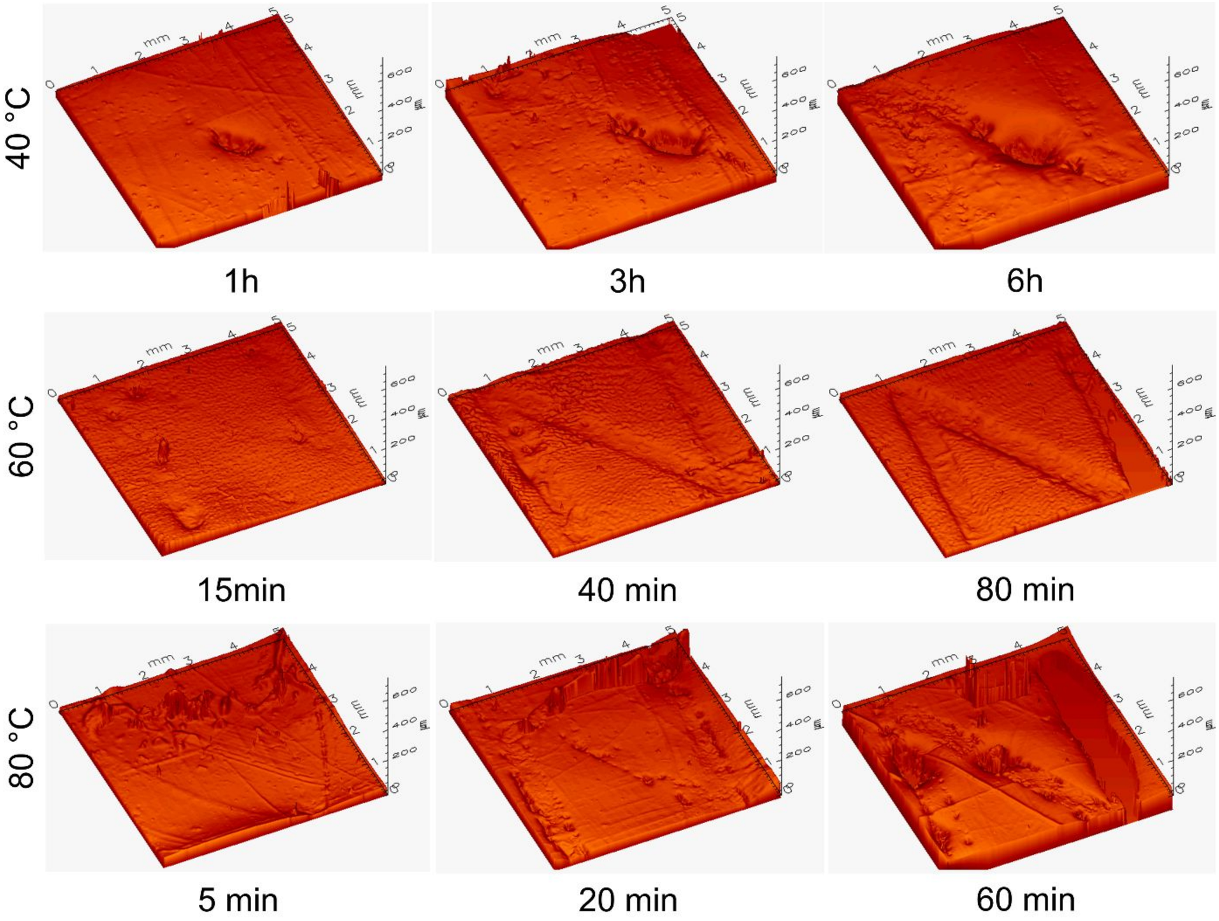

Figure S13. 3D plots of topology surface scans of the *dyn-0.5* material imprinted at different temperatures and times.
